# Evolution of the S-Genomes in *Triticum-Aegilops* Alliance: Evidences From Chromosome Analysis

**DOI:** 10.3389/fpls.2018.01756

**Published:** 2018-12-04

**Authors:** Alevtina S. Ruban, Ekaterina D. Badaeva

**Affiliations:** ^1^Laboratory of Chromosome Structure and Function, Leibniz Institute of Plant Genetics and Crop Plant Research (IPK), Gatersleben, Germany; ^2^Laboratory of Genetic Basis of Plant Identification, Vavilov Institute of General Genetics, Russian Academy of Sciences, Moscow, Russia; ^3^Laboratory of Molecular Karyology, Engelhardt Institute of Molecular Biology, Russian Academy of Sciences, Moscow, Russia

**Keywords:** wheat, *Aegilops*, S-genome of *Ae. speltoides*, S^*^-genome of other *Aegilops* species, chromosome, karyotype evolution, C-banding, FISH

## Abstract

Five diploid *Aegilops* species of the *Sitopsis* section: *Ae. speltoides, Ae. longissima, Ae. sharonensis, Ae. searsii*, and *Ae. bicornis*, two tetraploid species *Ae. peregrina* (= *Ae. variabilis*) and *Ae. kotschyi* (*Aegilops* section) and hexaploid *Ae. vavilovii* (*Vertebrata* section) carry the S-genomes. The B- and G-genomes of polyploid wheat are also the derivatives of the S-genome. Evolution of the S-genome species was studied using Giemsa C-banding and fluorescence *in situ* hybridization (FISH) with DNA probes representing 5S (pTa794) and 18S-5.8S-26S (pTa71) rDNAs as well as nine tandem repeats: pSc119.2, pAesp_SAT86, Spelt-1, Spelt-52, pAs1, pTa-535, and pTa-s53. To correlate the C-banding and FISH patterns we used the microsatellites (CTT)_10_ and (GTT)_9_, which are major components of the C-banding positive heterochromatin in wheat. According to the results obtained, diploid species split into two groups corresponding to *Emarginata* and *Truncata* sub-sections, which differ in the C-banding patterns, distribution of rDNA and other repeats. The B- and G-genomes of polyploid wheat are most closely related to the S-genome of *Ae. speltoides*. The genomes of allopolyploid wheat have been evolved as a result of different species-specific chromosome translocations, sequence amplification, elimination and re-patterning of repetitive DNA sequences. These events occurred independently in different wheat species and in *Ae. speltoides*_._ The 5S rDNA locus of chromosome 1S was probably lost in ancient *Ae. speltoides* prior to formation of Timopheevii wheat, but after the emergence of ancient emmer. Evolution of *Emarginata* species was associated with an increase of C-banding and (CTT)_10_-positive heterochromatin, amplification of Spelt-52, re-pattering of the pAesp_SAT86, and a gradual decrease in the amount of the D-genome-specific repeats pAs1, pTa-535, and pTa-s53. The emergence of *Ae. peregrina* and *Ae. kotschyi* did not lead to significant changes of the S^*^-genomes. However, partial elimination of 45S rDNA repeats from 5S^*^ and 6S^*^ chromosomes and alterations of C-banding and FISH-patterns have been detected. Similarity of the S^v^-genome of *Ae. vavilovii* with the S^s^ genome of diploid *Ae. searsii* confirmed the origin of this hexaploid. A model of the S-genome evolution is suggested.

## Introduction

Evolutionary goat grasses, or *Aegilops* are closely related to wheat and contributed two of the three subgenomes of hexaploid bread wheat (Sears, 1969; Kihara, 1975; Feldman, 2001). The natural distribution area of the genus *Aegilops* L. covers the Mediterranean basin, southwestern and central Asia (Witcombe, 1983; Kimber and Feldman, 1987; Van Slageren, 1994; Kilian et al., 2011). Their center of origin is thought to be located in Transcaucasia (Hammer, 1980; Van Slageren, 1994), or in the Fertile Crescent (Kimber and Feldman, 1987). These regions contain the highest concentration of *Aegilops* species. Goat grasses inhabit a broad range of environments and are characterized by very wide adaptation. Owing to this, many goat grasses exhibit good resistance to fungal diseases and pests (Hammer, 1980; Gill et al., 1985; Makkouk et al., 1994; El Bouhssini et al., 1998; Monneveux et al., 2000; Schneider et al., 2008; Zhao et al., 2016), heat, drought or frost tolerance and cold hardiness (Limin and Fowler, 1985; Damania et al., 1992; Monneveux et al., 2000; Pradhan et al., 2012). Some *Aegilops* accessions are characterized by high grain quality and increased micronutrient content (Rawat et al., 2011; Farkas et al., 2014; Rakszegi et al., 2017) that can be used for wheat improvement. Although many agronomically useful genes have already been transferred from *Aegilops* to common wheat varieties or breeding lines (Knott and Dvorák, 1976; Schneider et al., 2008; Rawat et al., 2011; McIntosh et al., 2013; Zhang et al., 2015), their genetic potential in broadening genetic diversity of wheat is not fully exploited. Utilization of gene pool of *Aegilops* requires good knowledge of genetics and genomics of these species, including their karyotypes and chromosomal structures.

In addition to the great potential for wheat breeding, goat grasses can also be an attractive model for studying mechanisms of reticulate evolution. Depending on taxonomical system, the genus *Aegilops* is classified into 20 (Kihara, 1954), 22 (Zhukovsky, 1928; Eig, 1929; Van Slageren, 1994), 24 (Kimber and Feldman, 1987), 25 (Chennaveeraiah, 1960), or 26 species (Witcombe, 1983). These species are split into sections based on morphological criteria or genome composition. At present, the system suggested by Van Slageren (1994) is commonly accepted; therefore, we will follow this nomenclature. According to it, 10 *Aegilops* species are diploid and 12 – polyploid, that were formed as a result of hybridization of different diploid progenitors.

Based on a series of pioneering works of the famous Japanese geneticist (Kihara, 1937, 1949, 1954, 1957, 1963; Lilienfeld, 1951; Kihara et al., 1959), diploid *Aegilops* were divided into three major genomic groups, C, D, and S. The C-genome group included two species; the D-genome group included four species; and the S-genome group consisted of three species of the *Sitopsis* (Jaub. & Spach) Zhuk. section: *Ae. longissima* Schweinf. & Muschl. (including *Ae. sharonensis* Eig), *Ae. bicornis* (Forssk.) Jaub. & Spach, and *Ae. speltoides* Tausch (Kihara, 1937, 1949; Lilienfeld, 1951). A new diploid species of the *Sitopsis* section—*Ae. searsii* Feldman and Kislev ex Hammer, has been discovered later by Feldman and Kislev (1977). Analysis of the karyotype, meiotic chromosome pairing, pollen fertility and seed set in *Ae. longissima x Ae. searsii* hybrids showed that *Ae. searsii* possesses the S^*^-genome (Feldman et al., 1979; Yen and Kimber, 1990a).

Thus, current taxonomy recognizes five diploid species carrying the S-genome: *Ae. speltoides* including ssp. *ligustica* (Savign.) Fiori (SS) and ssp. *speltoides* Boiss., *Ae. bicornis* (S^b^S^b^), *Ae. searsii* (S^s^S^s^), *Ae. sharonensis* (S^sh^S^sh^), and *Ae. longissima* (S^l^S^l^) (Van Slageren, 1994; Kilian et al., 2011; Feldman and Levy, 2015). These species are morphologically similar, but can be easily distinguished by their habitat, climatic adaptation, and distribution areas. Based on differences in spike morphology, Eig (1929) divided the *Sitopsis* group into two sub-sections, *Truncata* and *Emarginata*. Subsection *Truncata* includes only one species–*Ae. speltoides* (SS), which grows in central, eastern, and northern part of the *Sitopsis* area. This species consists of two forms, *ligustica* and *auscheri*, which differ in their fruiting spike and the mode of seed dispersal (Eig, 1929; Zohary and Imber, 1963), but are similar in karyotype structure (Chennaveeraiah, 1960). Their hybrids are fully fertile and show complete meiotic chromosome pairing (Zohary and Imber, 1963). *Ae. speltoides* has the lowest nuclear DNA content (1C = 5.81 ± 0.123 pg) within the *Sitosis* group (Eilam et al., 2007) and differs significantly from *Emarginata* species in its chromosome morphology (Chennaveeraiah, 1960), Giemsa C-banding (Teoh and Hutchinson, 1983; Friebe and Gill, 1996; Friebe et al., 2000) and FISH patterns (Yamamoto, 1992a,b; Jiang and Gill, 1994b; Badaeva et al., 1996a,b; Salina et al., 2006b; Raskina et al., 2011; Belyayev and Raskina, 2013).

The subsection *Emarginata* includes four species: *Ae. bicornis, Ae. searsii, Ae. sharonensis*, and *Ae. longissima*, which grow in the central and southern part of the *Sitopsis* section habitat (Feldman and Kislev, 1977). Study of the chromosome pairing of intraspecific hybrids (Kihara, 1954, 1963; Feldman et al., 1979; Yen and Kimber, 1989, 1990a,b,c), similarity of karyotype structure (Riley et al., 1958; Chennaveeraiah, 1960), the number and distribution of 5S and 45S rDNA loci (Yamamoto, 1992a,b; Badaeva et al., 1996b), and the distribution of pSc119.2 sequence (Badaeva et al., 1996a) suggest a close relationship of *Emarginata* species, although they differ from each other in genome size (Eilam et al., 2007) and C-banding patterns (Friebe and Gill, 1996).

Morphologically, *Ae. bicornis* is the most primitive species in this group (Eig, 1929). It is more difficult to produce hybrids with *Ae. bicornis* than with other *Aegilops* of the S-genome group (Kimber and Feldman, 1987). Genome size of *Ae. bicornis* (1C = 6.84 ± 0.097 pg) is only little larger than that of *Ae. searsii* (1C = 6.65 ± 0.091 pg), and is lower than of *Ae. longissima* (1C = 7.48 ± 0.082 pg) or *Ae. sharonensis* (1C = 7.52 ± 1.000 pg) (Eilam et al., 2007). Morphologically *Ae. searsii* resembles *Ae. longissima*, but differs from it in a number of morphological traits which are considered as evolutionary advanced (Feldman and Kislev, 1977). *Ae. longissima* x *Ae. searsii* hybrids exhibit meiotic irregularities and are highly sterile (Feldman et al., 1979). By contrast, the F_1_ hybrids *Ae. longissima* x *Ae. sharonensis* are fertile and show complete chromosome pairing in meiosis. Isolation of these species is caused by different ecological requirements (Feldman and Levy, 2015). According to other hypothesis (Waines and Johnson, 1972), *Ae. sharonensis* could be a hybrid between *Ae. longissima* and *Ae. bicornis*. *Ae. longissima* carries a species-specific 4S^*^/7S^*^ translocation (Tanaka, 1955; Yen and Kimber, 1990b; Friebe et al., 1993; Naranjo, 1995), while no structural rearrangements have been identified in other species of this group (Yen and Kimber, 1989, 1990a,b,c; Maestra and Naranjo, 1997, 1998; Luo et al., 2005; Dobrovolskaya et al., 2011).

The similarity of *Emarginata* species and separate position of *Ae. speltoides* within the *Sitopsis* section was confirmed by molecular analyses of nuclear and cytoplasmic DNA. Based on the variation of repeated nucleotide sequences (RNS) Dvorák and Zhang (1992) showed that the *Sitopsis* species are phylogenetically similar, but *Ae. speltoides* is clearly separated from species of the *Emarginata* group. RAPD- and AFLP analyses revealed that *Ae. speltoides* forms a cluster with polyploid wheats, which is separated from other *Sitopsis* species (Kilian et al., 2007, 2011; Goryunova et al., 2008). Study of organellar DNAs by PCR-single-strand conformational polymorphism (PCR-SSCP) revealed high similarity of *Ae. bicornis - Ae. sharonensis - Ae. longissima* plasmons and their distinctness from plasmon of *Ae. speltoides* (Wang et al., 1997).

Comparative sequence analysis provided further insights into the evolution of *Triticum* and *Aegilops* and allowed the estimation of divergence time of different genomic groups. Comparison of chloroplast (Yamane and Kawahara, 2005; Golovnina et al., 2007; Gornicki et al., 2014; Middleton et al., 2014; Bernhardt et al., 2017) and nuclear DNA sequences (Petersen et al., 2006; Salse et al., 2008; Marcussen et al., 2014) strongly suggest that *Ae. speltoides* occupies a basal position on the phylogenetic tree of *Aegilops/Triticum* (Petersen et al., 2006; Kawahara, 2009). Probably *Ae. speltoides* diverged from the progenitor of the Triticeae much earlier than diploid wheat and *Aegilops* species (Yamane and Kawahara, 2005; Salse et al., 2008; Gornicki et al., 2014; Middleton et al., 2014; Bernhardt et al., 2017). Estimates obtained from the analyses of nuclear DNA sequences placed the possible divergence time within the period from ~7 MYA (Marcussen et al., 2014) to 3.5–2.7 MYA (Dvorák and Akhunov, 2005; Salse et al., 2008). Estimates obtained from chloroplast DNA favored a more recent origin of *Ae. speltoides* – 4.1–3.6 MYA (Bernhardt et al., 2017) to 2.67 ± 1.1. MYA (Middleton et al., 2014). Marcussen et al. (2014) supposed that the D-genome lineage (which indeed included D, M, and S^*^ genome species, Sandve et al., 2015) emerged through ancient homoploid hybridization between A and S genomes. The members of *Emarginata* group are thought to radiate from common ancestor approximately 1.0–2.0 MYA (*Ae. searsii*) – 1.4 MYA (*Ae. bicornis*) – to 0.4 MYA (*Ae. sharonensis*) (Marcussen et al., 2014; Feldman and Levy, 2015).

Hypothesis that the B and G genomes of polyploid wheats originated from a diploid S-genome *Aegilops* species was put forward in the middle XX^th^ (Sears, 1956; Riley et al., 1958). Different taxa were suggested as potential progenitors of polyploid wheat (Haider, 2013). All species of the *Sitopsis* section have been considered as the B-genome donors: *Ae. speltoides* (Sarkar and Stebbins, 1956; Tanaka et al., 1979; Bahrman et al., 1988; Kerby et al., 1990; Daud and Gustafson, 1996; Maestra and Naranjo, 1998; Yan et al., 1998; Blake et al., 1999; Rodríguez et al., 2000a; Haider, 2013), *Ae. bicornis* (Sears, 1956), *Ae. longissima* (Tanaka, 1956; Konarev et al., 1976; Konarev, 1980; Peacock et al., 1981), *Ae. searsii* (Feldman and Kislev, 1977; Nath et al., 1983, 1984; Kerby et al., 1990; Liu et al., 2003), *Ae. sharonensis* (Kushnir and Halloran, 1981) or yet unknown species of the *Emarginata* group (Kerby et al., 1990). Molecular analyses of common wheat genome and genomes of related species confirmed the ancestry of wheat B- genome from *Ae. speltoides* or the species close to it (Talbert et al., 1991; Petersen et al., 2006; Goryunova et al., 2008; Salse et al., 2008; Marcussen et al., 2014). Based on the analysis of nuclear or plastid DNA, ancient tetraploid emmer could emerge 0.4–0.8 MYA (Huang et al., 2002; Dvorák and Akhunov, 2005; Yamane and Kawahara, 2005; Golovnina et al., 2007; Gornicki et al., 2014; Marcussen et al., 2014; Middleton et al., 2014; Bernhardt et al., 2017).

The origin of the G-genome of *Triticum timopheevii* Zhuk. from the S-genome of *Ae. speltoides* was first hypothesized by Giorgi and Bozzini (1969) based on comparison of chromosome morphologies and was later confirmed by numerous studies including chromosome pairing analysis of intraspecific hybrids (Shands and Kimber, 1973; Tanaka et al., 1979; Maestra and Naranjo, 1999; Rodríguez et al., 2000a), comparison of C-banding (Badaeva et al., 1996a) and ISH patterns (Jiang and Gill, 1994a,b; Salina et al., 2006b), isozyme profiles (Konarev et al., 1976; Nakai, 1978; Jaaska, 1980), AFLP- (Kilian et al., 2007, 2011) and RFLP-analyses (Dvorák and Zhang, 1990; Talbert et al., 1991; Dvorák, 1998), sequencing of nuclear (Huang et al., 2002) and cytoplasmic DNA (Sasanuma et al., 1996; Yamane and Kawahara, 2005; Golovnina et al., 2007; Gornicki et al., 2014). These studies revealed that *Ae. speltoides* is more closely related to the G genome of *T. timopheevii* than to the B-genome of common wheat and suggested that ancient *T. timopheevii* could emerge approximately 0.4 MYA (Huang et al., 2002; Gornicki et al., 2014).

The S^*^-genome is identified in two tetraploid *Aegilops* species belonging to the section *Aegilops* L.: *Ae. peregrina* (Hach. in Fraser) Maire & Weiller (= *Ae. variabilis* Eig, U^p^U^p^S^p^S^p^) and *Ae. kotschyi* Boiss. (U^k^U^k^S^k^S^k^). Based on the “analyzer” method H. Kihara (1954) proposed that *Ae. peregrina* is a hybrid between *Ae*. *umbellulata* Zhuk. and a diploid species of the *Sitopsis* group (Lilienfeld, 1951), although conventional chromosome staining did not reveal the S^*^-genome in these species (Chennaveeraiah, 1960). Cytoplasmic genomes of *Ae. peregrina* and *Ae. kotschyi* are most closely related to the cytoplasmic genome of *Ae. searsii* (Ogihara and Tsunewaki, 1988; Siregar et al., 1988). However, meiotic analysis of the F_1_ hybrids between *Ae. kotschyi* and induced autotetraploid of three *Sitopsis* species showed that *Ae. kotschyi* shared the S^*^ genome with *Ae. longissima* (Yen and Kimber, 1990d). Yu and Jahier (1992) come to the same conclusion based on chromosome pairing analysis in hybrids of *Ae. variabilis* (= *Ae. peregrina*) with different *Sitopsis* species. RFLP profiles of RNS suggested that the S^*^ genome of *Ae. peregrina* and *Ae. kotschyi* could have originated from *Ae. longissima* or *Ae. sharonensis* or the species immediately preceding the divergence of these diploids (Zhang et al., 1992). C-banding and FISH analyses confirmed highest similarity of the S^*^-genome of these tetraploids with *Ae. longissima* or *Ae. sharonensis* (Jewell, 1979; Jewell and Driscoll, 1983; Friebe et al., 1996; Badaeva et al., 2004; Zhao et al., 2016).

*Ae. vavilovii* (Zhuk.) Chennav. (D^1^D^1^X^*cr*^X^cr^S^v^S^v^) is a hexaploid taxa belonging to section *Vertebrata* Zhuk. emend Kihara, complex Crassa. *Ae. vavilovii* originated from hybridization of tetraploid *Ae. crassa* Boiss. with a species of *Emarginata* group, possibly *Ae. longissima* (Kihara, 1963; Kihara and Tanaka, 1970). Originally *Ae. vavilovii* was treated as a subspecies of hexaploid *Ae. crassa*, and its taxonomic rank was raised to independent biological species by Chennaveeraiah (1960). Although this author was unable to determine genome constitution of *Ae. vavilovii*, he noticed a pairwise similarity of the satellite chromosomes in karyotype of this species.

Yen and Kimber (1992) failed to identify the exact donor of the S^v^-genome of *Ae. vavilovii* based on analysis of chromosome pairing in the F_1_ hybrids of *Ae. vavilovii* with induced autotetraploids of the *Sitopsis* species and proposed that the S^v^-genome is substantially modified. By using molecular markers (Talbert et al., 1991) showed that the S^v^-genome of *Ae. vavilovii* is related to the S^*^-genome of *Emarginata* group. Data collected by molecular methods (Zhang and Dvorák, 1992), C-banding and FISH analyses (Badaeva et al., 2002; Zhang et al., 2002) confirmed, that *Ae. vavilovii* contains the S^v^-genome that could probably derive from *Ae. searsii* (Badaeva et al., 2002).

Because of the genetic relatedness of the S-genome *Aegilops* species and polyploid wheats as well as of their potential for wheat improvement, they have been attracting the attention of researchers over the past century. Numerous intraspecific hybrids have been created to transfer desired genes from *Aegilops* to wheat (Schneider et al., 2008). Sets of addition, substitution or translocation wheat-*Aegilops* lines, including *Ae. speltoides* (Friebe et al., 2000; Liu et al., 2016), *Ae. searsii* (Pietro et al., 1988; Friebe et al., 1995), *Ae. sharonensis* (Olivera et al., 2013), *Ae. longissima* (Friebe et al., 1993), and polyploid *Ae. peregrina*, (Jewell and Driscoll, 1983; Friebe et al., 1996; Yang et al., 1996) and *Ae. kotschyi* (Rawat et al., 2011) were obtained and characterized using a combination of C-banding and analyses with the group-specific molecular or isozyme markers. As a result of these studies, the genetic classifications were developed for C-banded chromosomes of several S-genome species (Friebe and Gill, 1996).

From another side, the S-genomes were extensively examined by FISH with various DNA probes (Yamamoto, 1992a; Badaeva et al., 1996a,b, 2002, 2004; Belyayev et al., 2001; Zhang et al., 2002; Giorgi et al., 2003; Salina et al., 2006b, 2009; Raskina et al., 2011; Ruban et al., 2014; Molnár et al., 2016; Zhao et al., 2016). Probe pSc119.2 was used most frequently (Badaeva et al., 1996a, 2002, 2004; Molnár et al., 2016; Zhao et al., 2016), however, the pSc119.2 signals are located predominantly in subtelomeric chromosome regions, thus hindering unequivocal chromosome identification. Probe pAs1, which proves to be highly informative for many *Aegilops* species, is not very useful for the S-genome analysis owing to a small number of detected sites (Badaeva et al., 1996a). In most papers FISH-labeled *Aegilops* chromosomes were classified based on their morphology, which is not sufficient to determine their correspondence to the genetic nomenclature of C-banded chromosomes. Owing to this, it was necessary to find FISH markers for the precise identification of all S-genome chromosomes and coordination of classification systems.

Recently, Komuro et al. (2013) isolated and characterized a number of repetitive DNAs from the wheat genome, which can potentially be used for molecular-cytogenetic analysis of wheat and *Aegilops* species. Several new sequences have been described in other papers (Salina et al., 1998, 2009; Adonina et al., 2015; Badaeva et al., 2015; Zhao et al., 2016). In this study we characterized the S genomes of diploid and polyploid *Triticum* and *Aegilops* species using C-banding and FISH with a set of “classical” [pSc119.2, pAs1, pTa71, pTa794, Spelt-1, Spelt-52] and novel [pAesp_SAT86, (CTT)_n_, (GTT)_n_, pTa-535, pTa-s53] probes in order to assess evolutionary changes in the *Triticum-Aegilops* alliance.

## Materials and Methods

### Plant Material

Five diploid (*Aegilops speltoides, Ae. longissima, Ae. sharonensis, Ae. searsii, Ae. bicornis*), two tetraploid (*Ae. peregrina* and *Ae. kotschyi*) and one hexaploid (*Ae. vavilovii*) *Aegilops* species carrying the S-genome have been examined in comparison with two tetraploid wheats, *T. timopheevii* and *T. dicoccoides*. The list of accessions, their ploidy level, genome constitution and the origin are given in Table S1.

### DNA Probe*s*

Following probes were used for FISH:

Plasmid clones pTa71 - a 9 kb long sequence of common wheat encoding 18S, 5.8S and 26S rRNA genes including spacers (Gerlach and Bedbrook, 1979), pTa794 – a 420 bp long sequence of wheat containing the 5S rRNA gene and intergenic spacer (Gerlach and Dyer, 1980), pAs1 - a 1 kb fragment derived from *Ae. tauschii* and belonging to *Afa* family (Rayburn and Gill, 1986), pSc119.2 – a 120 bp long sequence isolated from rye (Bedbrook et al., 1980), pTa-s53 – a 587 bp DNA fragment isolated from common wheat (Komuro et al., 2013), Spelt-1 – a 150 bp fragment isolated from *Ae. speltoides* (Salina et al., 1997), Spelt-52 (homolog of pAesKB52) – a 276 bp long DNA fragment isolated from *Ae. speltoides* (Salina et al., 2004a), and pAesp_SAT86 - a new satellite family with a monomer length of 86 bp isolated from Ae. *speltoides* genomic DNA (Badaeva et al., 2015) and showing 91-94% similarity to wheat repeat pTa-713 described in Komuro et al. (2013) were labeled with dUTP-ATTO-488, dUTP-ATTO-550, dUTP-ATTO-647N by nick-translation using an Atto NT Labeling Kit (Jena Bioscience, Germany) or with FITC (fluorescein-12-dUTP, Roche, Germany) or biotin (biotin-16-dUTP, Roche, Germany) by nick-translation using the Nick Translation Mix (Roche, Germany) according to manufacturers' instruction.

Probe pTa535-1 was used as 5′ 6-carboxyfluorescein (6-FAM) or 6-carboxytetramethylrhodamine (TAMRA) end-labeled (MWG, Germany) oligo probe (5′-AAA AAC TTG ACG CAC GTC ACG TAC AAA TTG GAC AAA CTC TTT CGG AGT ATC AGG GTT TC-3′) (Komuro et al., 2013; Tang et al., 2014).

The oligo-(CTT)_10_ or complementary oligo-(GAA)_10_ probes [thereafter (CTT)_n_] were labeled with 5/6-Sulforhodamine 101-PEG3-Azide or 6-Carboxyfluorescein Azide by click chemistry (Baseclick, Germany).

The oligo-(GTT)_9_ probe labeled at the 3′-end with fluorescein-12-dUTP was synthesized in the Laboratory of Biological Microchips at the Engelhardt Institute of Molecular Biology, Moscow, Russia.

### Giemsa C-Banding Method

The Giemsa C-banding method described in Badaeva et al. (1994) was used for analysis. Seeds were soaked in water for 24 h at room temperature and then kept at 4°C overnight on wet filter paper in Petri dishes. For the next 24 h Petri dishes were placed at 24°C. Roots were cut and treated with 0.05% colchicine for 3 h. Further, roots were fixed in 45% acetic acid for 4 h, washed with distilled water and treated with 0.2 N HCl for 15 min at 4°C and for 5 min at 60°C. After overnight treatment with a 4 mg/ml Cellulysine (Fluka, Switzerland) solution at 24°C root meristems were squashed in drop of 45% acetic acid. Slides were frozen in liquid nitrogen and coverslips were removed. After that slides were placed into 96% ethanol at room temperature. Chromosomes of wheat were classified according to nomenclature suggested in Gill et al. (1991), Badaeva et al. (2016); chromosomes of *Aegilops* species were classified according to the nomenclature of Friebe et al. (1993, 1995, 1996, 2000), Friebe and Gill (1996). Karyotype of one typical accession per each species was taken as standard for alignment of C-banding and FISH patterns.

### Fluorescence *in situ* Hybridization

Detailed protocols of the pretreatment of the materials, fixation and chromosomal preparation are given in Badaeva et al. (2017). Briefly, seeds were germinated in Petri dishes on wet filter paper at 24°C in dark. Roots were excised when 2 cm long, treated with ice-cold water for 24 h, and fixed with ethanol:acetic acid (3:1) fixative for at least 4 days at room temperature. Before slide preparation roots were stained in 2% acetocarmine for 15 min. Meristems were cut off and squashed in a drop of 45% acetic acid. Slides were frozen in liquid nitrogen and coverslips were removed with a razor blade. The slides were kept in 96% ethanol in a freezer.

Hybridization mixture contained 1 g dextran sulfate dissolved in 1 ml of distilled water, 5 ml deionized formamide, 1 ml of 20x SSC, 1 ml Herring sperm DNA (10 mg/ml, Promega, USA). Per slide 40–60 ng of each labeled probe were added to 18 μl hybridization mixture. Post hybridization washes were carried out as follows: for probes labeled with biotin or fluorescein the slides were washed in 0.1x SSC 2 × 10 min, then in 2x SSC 2 × 10 min at 42°C. Slides hybridized with directly labeled probes were washed at 58°C in 2x SSC for 20 min. The probes labeled with fluorescein were detected using anti-fluorescein/Oregon green®, rabbit IgG fraction, Alexa Fluor® 488 conjugated antibody (Molecular Probes, USA). Biotin was detected with sptreptavidin-Cy3 (Amersham Pharmacia Biotech, USA). The slides were counter-stained with DAPI (4′,6-diamidino-2-phenylindole) in Vectashield mounting media (Vector laboratories, Peterborough, UK) and examined with a Zeiss Imager D-1 microscope. Selected metaphase cells were captured with an AxioCam HRm digital camera using software AxioVision, version 4.6. Images were processed in Adobe Photoshop^R^, version CS5 (Adobe Systems, Edinburgh, UK). For classification, chromosomes were aligned with the C-banding patterns based on the hybridization patterns of labeled CTT- and GTT-satellite sequences.

## Results

### Analysis of Diploid Species

According to the C-banding and FISH patterns of nine probes, five diploid species of the *Sitopsis* section split into two groups corresponding to taxonomically recognized sub-sections *Truncata* (*Ae. speltoides*) and *Emarginata* (*Ae. longissima, Ae. sharonensis, Ae. searsii, Ae. bicornis*).

#### Sub-section *Truncata: Ae. speltoides*

The karyotype of *Ae. speltoides* consists of metacentric or submetacentric chromosomes; the chromosome pairs 1S and 6S carry large satellites in their short arms (Figure S1). All chromosomes contain large Giemsa-positive pericentromic heterochromatin, prominent subtelomeric C-bands, and some small or medium sized interstitial bands. Giemsa-patterns allowed the identification of all *Ae. speltoides* chromosomes. We observed significant variations of Giemsa bands between plants within and between accessions. Heteromorphism of homologous chromosome has been recorded in all studied genotypes (Figure S1).

**Figure 1:**
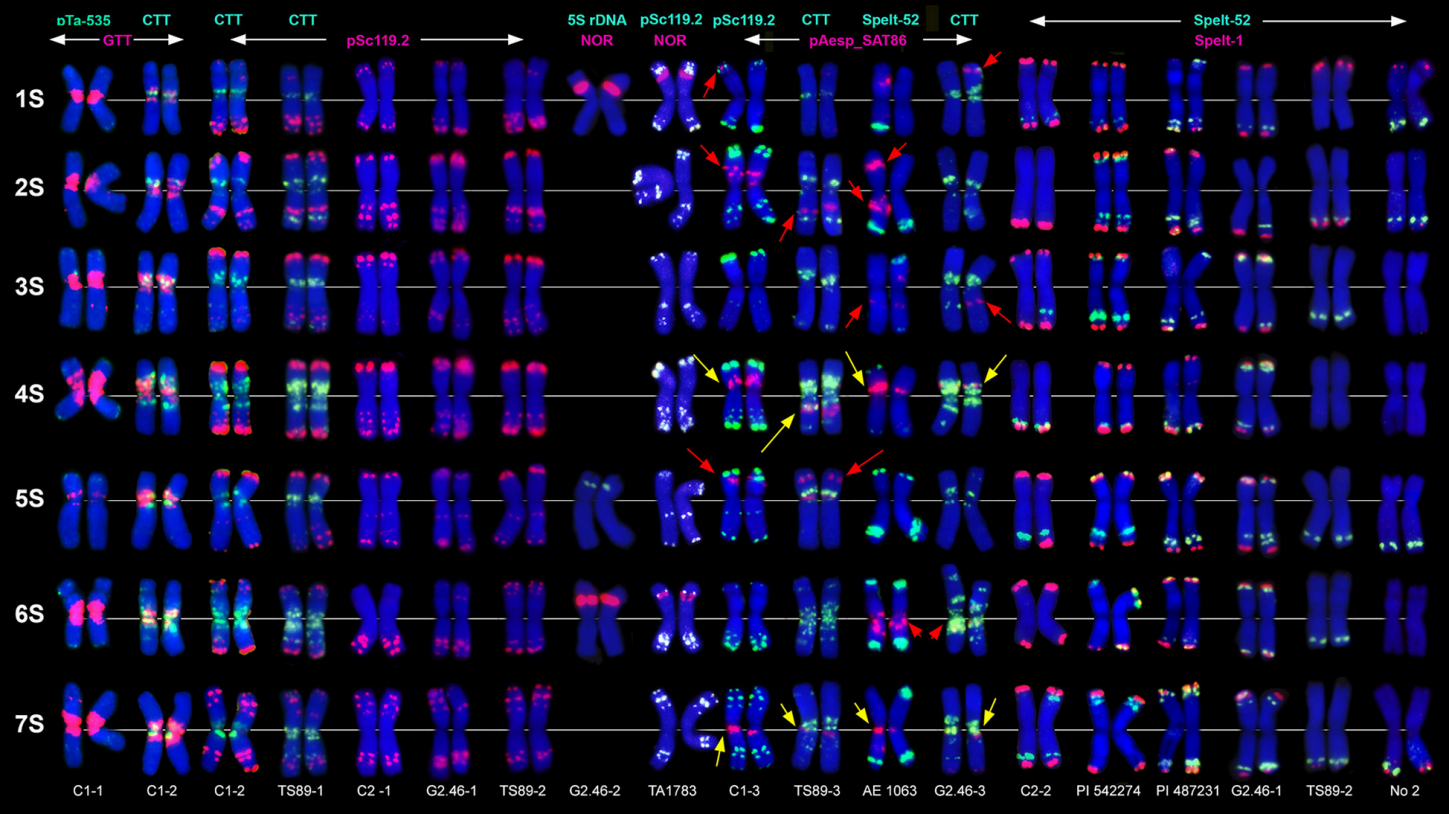
Localization of different DNA sequences on chromosomes of *Ae. speltoides*. Probe combinations are shown on the top; signal color corresponds to probe name. Accessions numbers are indicated in the bottom: C1-1–C1-3 genotypes from Technion park, Haifa, Israel; TS89-1–TS89-3–genotypes from Katzir, Israel; C2-1–C2-2–genotypes from Nahal Mearot, Israel; G2.46-1–G2.46-3–genotypes from Ramat haNadiv, Israel. Permanent pAesp_SAT86 loci are indicated with yellow arrows; polymorphic sites are shown with red arrows.

**Figure 2:**
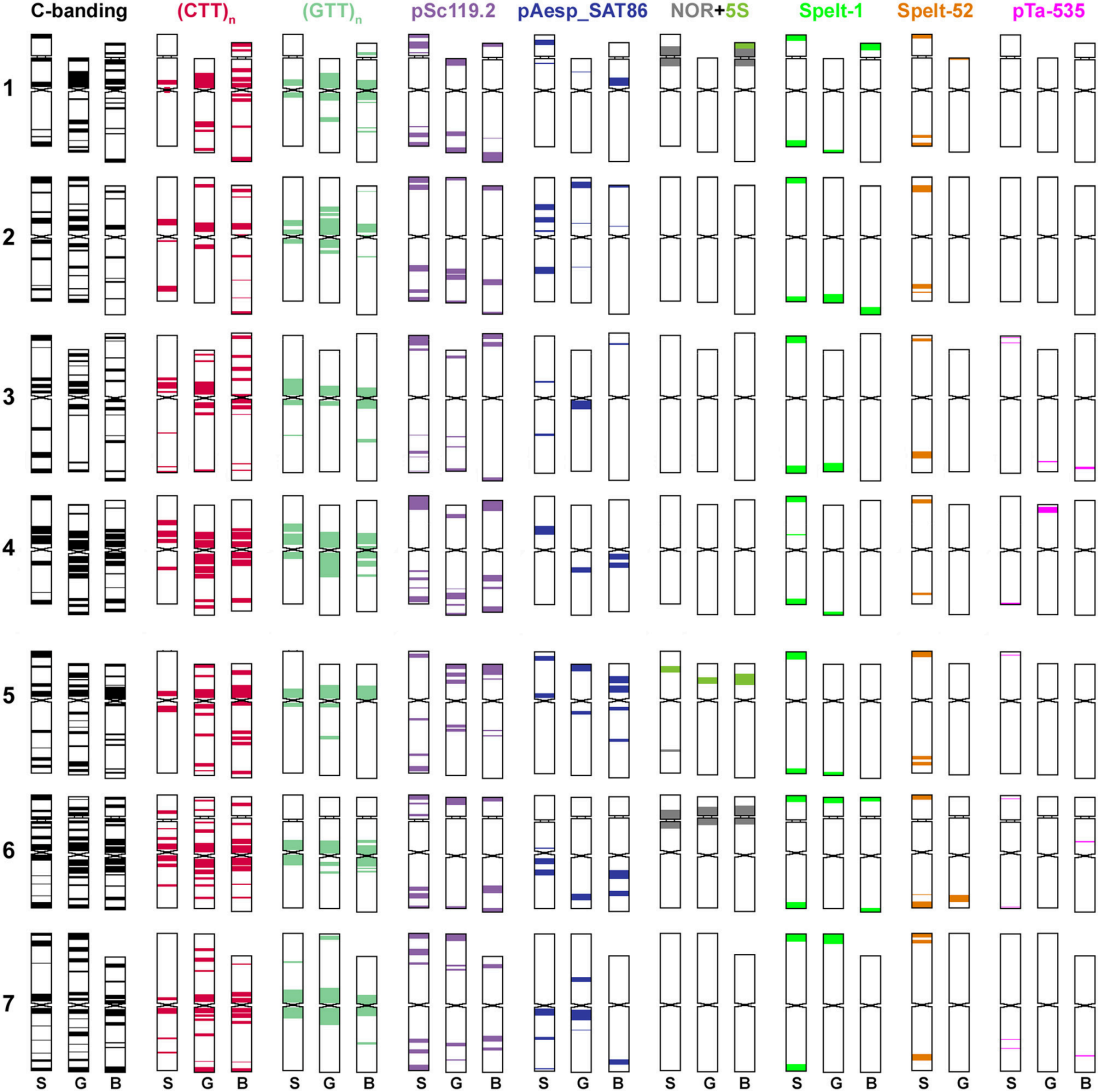
Idiogram showing relative positions of C-bands and nine DNA probes (probe names are given on the top) on chromosomes of *Ae. speltoides* (S), the B-genome (B) of common wheat and G-genome (G) of *T. timopheevii*.

**Figure 3:**
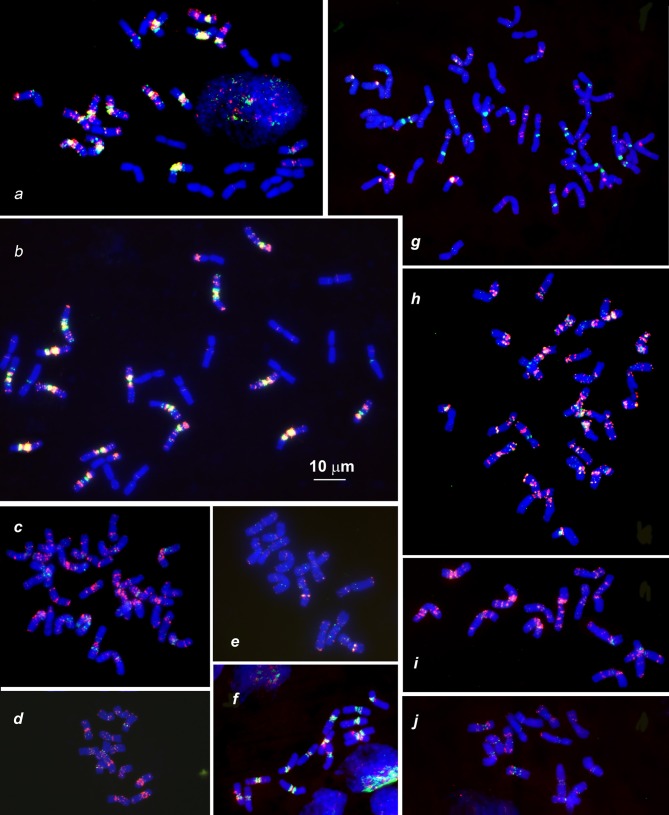
Distribution of (CTT)_10_ and (GTT)_9_ probes (red and green colors respectively) on chromosomes of wheat and *Aegilops* species: **(a)**, *Triticum dicoccoides* (IG 46396); **(b)**, *T. araraticum* (IG 116164); **(c)**, *Ae. kotschyi* (TA2206); **(d)**, *Ae. longissmia* (AE 904); **(e)**, *Ae. searsii* (AE 1071); **(f)**, *Ae. speltoides* (C1, Technion park, Israel); **(g)**, *Ae. vavilovii* (K-3637); **(h)**, *Ae. peregrina* (C11, Nahal Mearot, Israel); **(i)**, *Ae. sharonensis* (C6, Keshon, Israel); **(j)**, *Ae. bicornis* (K-666). Scale bar, 10 μm.

The (CTT)_10_ clusters (Figure 1, CTT) are located in proximal and interstitial chromosome regions, overlapping with Giemsa N-bands (Jiang and Gill, 1994b). No (CTT)_10_ signals were found in the sub-telomeric parts of the chromosomes possessing C-bands. The (GTT)_9_ probe forms prominent proximal clusters (Figure 1, GTT, Figures 2, 3f), often exceeding the size of (CTT)_n_-signals. The abundance of the GTT-microsatellite is an important diagnostic feature of *Ae. speltoides* chromosomes.

The pSc119.2 labeling patterns are represented by subtelomeric and interstitial signals allowing the discrimination of all *Ae. speltoides* chromosomes. Some hybridization sites are found in all genotypes, whereas other vary in the presence and signal size (Figure 1). Based on dual-color FISH with (CTT)_10_ and pSc119.2 probes we corrected previously published classification of pSc119.2-labeled chromosomes (Badaeva et al., 1996a) according to the genetic nomenclature (Friebe et al., 2000). In particular, the chromosomes 2S and 3S have been renamed.

Major NORs are detected on chromosomes 1S and 6S, and one pair of 5S rDNA loci are mapped on the chromosome 5S (Figures 1, 2). In addition, accession TA1873 shows one minor site on the long arm of one 5S chromosome.

Repeat pAesp_SAT86 exhibits significant variation of labeling patterns between *Ae. speltoides* genotypes (Figure 1). Two sites located in the short arm of 4S and pericentromeric region of 7SL are permanent (Figure 1, yellow arrows). In genotypeTS89 this repeat is transferred to the long arm of 4S, probably due to a pericentric inversion. Several facultative pAesp_SAT86 sites were found in more than one genotype (Figure 1, red arrows), while some signals were detected in single genotypes on either one or both homologous chromosomes.

The labeling patterns of Spelt-1 and Spelt-52 probes are highly polymorphic (Figure 1). The Spelt-1 sequence is located in subtelomeric regions of either one or two chromosome arms. The number of loci per diploid genome varied from six (TS89 Katzir and No2 from Turkey) to 27 (PI 542274 from Turkey). Genotypes differ from each other in the size and chromosome location of the Spelt-1 clusters. The Spelt-52 signals of variable size are located in distal chromosome regions, proximally to Spelt-1. The number of Spelt-52 clusters per diploid genome varied from eight to 22 (Figure 1), the size and chromosomal distribution are highly polymorphic. Genotypes differ from each other in a ratio of Spelt-1/Spelt-52 repeats. Thus, the Spelt-1 could significantly prevail over Spelt-52, or the Spelt-52 could be more abundant (Figure 1).

Only few inconsistent, dot-like pTa-535 signals have been detected in *Ae. speltoides* (Figure 1). No hybridization was found with pAs1 and pTa-s53 probes.

#### Sub-section Emarginata

Four species of the *Emarginata* sub-section have a similar karyotype, which is distinct from that of *Ae. speltoides* (Figures S1, S2). Chromosome pairs 5S^*^ and 6S^*^ carry unequal satellites: large on 6S^*^ and small on 5S^*^ chromosomes (Figure S2). Most *Ae. sharonensis* genotypes collected in Keshon (Israel) are heterozygotes (Figures S2c1,c2) indicating that open pollination is common in this population.

The karyotypes of *Emarginata* species differ in heterochromatin content detected by Giemsa staining. *Ae. bicornis* and *Ae. searsii* showed small-to-medium C-bands located in interstitial chromosome regions (Figures S2a1–b4). *Ae. sharonensis* and *Ae. longissima* exhibit prominent pericentromeric and subtelomeric and many interstitial C-bands (Figures S2c1–d10). C-banding patterns allowed the chromosome identification in all *Emarginata* species. A species-specific translocation between 4S^*^ and 7S^*^ is found in all *Ae. longissima* accessions.

**Figure 4:**
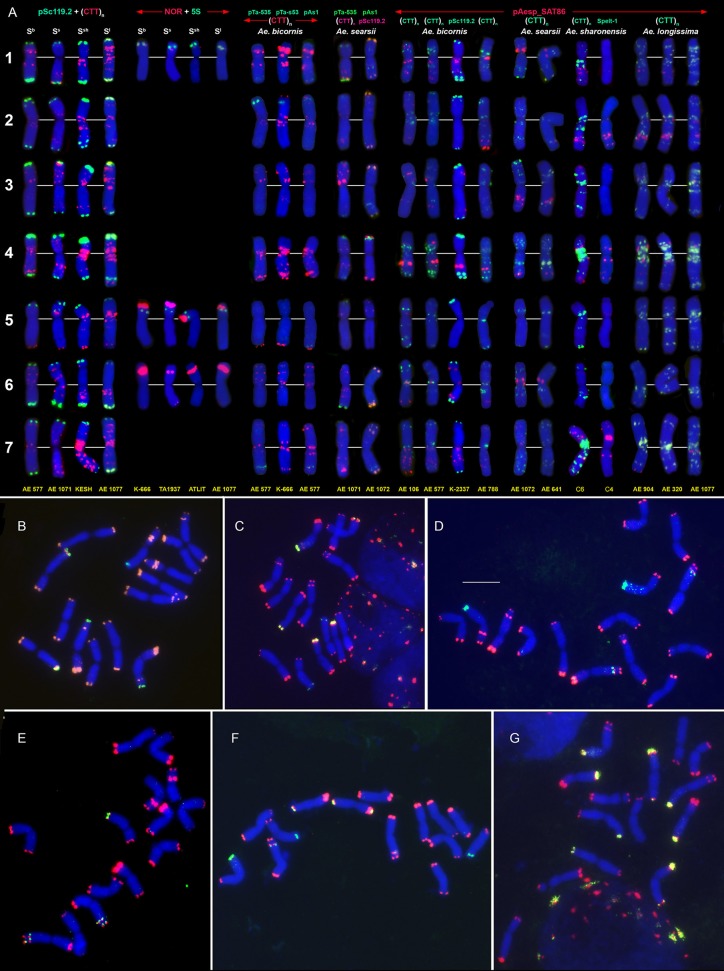
Distribution of repeated DNA sequences on chromosomes of four species of *Emarginata* group **(A)**, *Ae. bicornis* (S^b^), *Ae. searsii* (S^s^), *Ae. sharonensis* (S^sh^), and *Ae. longissima* (S^l^). **(A)** Probe combinations are given on the top, accession names are shown below karyograms. Signal color corresponds to probe name. 1–7 – homoeologous groups. Polymorphisms of Spelt-52 patterns on *Ae. longissima* **(B–D)** and *Ae. sharonensis* **(E–G)** chromosomes: **(B)**, K-905; **(C)**, K-907; **(D)**, C3 (HaBonim); **(E)**, C6 (Keshon); **(F)**, C7 (HaBonim); **(G)**, i-570030. The pSc119.2 signals are shown in red, Spelt-52–in green. Scale bar, 10 μm.

**Figure 5:**
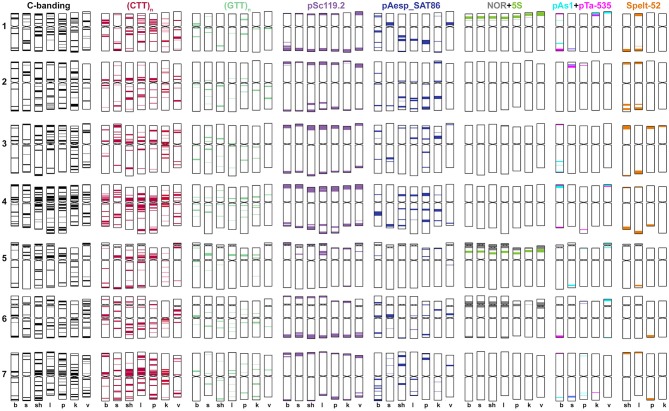
Idiogram showing relative positions of C-bands and nine DNA probes (probe names are given on the top) on the S*genome chromosomes of *Ae. bicornis* (b), *Ae. searsii* (s), *Ae. sharonensis* (sh), *Ae. longissima* (l), *Ae. peregrina* (p), *Ae. kotschyi* (k), and *Ae. vavilovii* (v).

The (CTT)_10_-hybridization pattern (Figures 3d,e,i,j, 4, 5) corresponds to the C-banding pattern. As expected, *Ae. bicornis* and *Ae. searsii* carry predominantly small CTT-signals (Figures 3e,j), while *Ae. sharonensis* and *Ae. longissima* possess prominent pericentromeric and distinct interstitial CTT-clusters. In contrast to *Ae. speltoides*, the (GTT)_9_ probe hybridizes poorly on the chromosomes of *Emarginata* species. Probably, accumulation of heterochromatin in this evolutionary lineage was mainly due to amplification of CTT-repeat, contributing to an increase of nuclear DNA content in *Ae. sharonensis/Ae. longissima* genomes as compared to *Ae. bicornis/Ae. searsii* (Eilam et al., 2007).

*Emarginata* species display similar pSc119.2 hybridization patterns consisting of subtelomeric signals of variable size in one or both chromosome arms. Interstitial loci were rarely found (Figures 4, 5). Permanent interstitial sites are found on 2S^*^S (*Ae. sharonensis* and *Ae. bicornis*), 4S^l^L (*Ae. longissima*), and 7S^s^ (*Ae. searsii*) only. The pSc119.2 cluster in the middle of 5S^*^S is present in all *Ae. searsii* accessions and some *Ae. longissima* and *Ae. sharonensis* lines (Figure 4). One or two polymorphic pSc119.2 sites were rarely observed on 1S^b^L and 4S^b^L of *Ae. bicornis*.

The number and location of 5S and 45S rDNA loci in *Emarginata* species is similar and differ from that in *Ae. speltoides* (Figures 1, 4). Major NORs are located on 5S^*^S and 6S^*^S and permanent minor NORs are found on 1S^*^S (Figure 4). Additional minor sites were detected in the terminal region of 6S^*^L of all *Ae. searsii* accessions and some lines of *Ae. bicornis* and *Ae. longissima*. All species possess two 5S rDNA loci located in the short arms of chromosome 1S^*^ and 5S^*^, distally (1S^*^) or proximally (5S^*^) to the 45S rDNA loci.

The distribution of pAesp_SAT86 signals is species- and chromosome-specific. An intraspecific polymorphism was detected in *Ae. bicornis* and *Ae. longissima*, labeling patterns are virtually invariable in *Ae. searsii* (Figures 4, 5). Distribution of pAesp_SAT86 clusters on *Ae. sharonensis* and *Ae. longissima* chromosomes is similar and differs from *Ae. bicornis* and *Ae. searsii*, which, in turn, are clearly distinct from each other. No similarity between homoeologous chromosomes of different species has been observed, though the chromosome 3S^s^ (*Ae. searsii*) shows almost the same distribution of pAesp_SAT86 sequence as the chromosomes 2S^*^ of *Ae. sharonensis* and *Ae. longissima*.

The Spelt-1 repeat was not found in any *Emarginata* species, and Spelt-52 is detected in *Ae. sharonensis* and *Ae. longissima* only (Figures 4B–G). Signals of variable size are located in terminal regions of either one or both arms of all chromosomes except 6S^*^. Only two interstitial loci are found in the long arms of 2S^*^ and 4S^*^. Distribution of Spelt-52 is highly diverse and polymorphisms are often observed even between homologous chromosomes. Depending on genotype, the number of signals ranges from 0 to 14. Most *Ae. longissima* accessions carry a Spelt-52 site in the long arm of 5S^*^, while it is absent in six out of 8 *Ae. sharonensis* accessions (Table S2). No other differences in labeling patterns were found between these species.

Distinct signals of the D-genome specific probes pAs1, pTa-535 or pTa-s53 are revealed in *Ae. bicornis* and *Ae. searsii* only (Figures 4A, 5). Two small pTa-535 sites are found in the distal parts of 2S^b^S and 7S^b^L chromosomes of *Ae. bicornis;* the first one overlaps with pTa-s53, and the second—with pAs1 sites. The pTa-535 probe hybridizes to subterminal regions of five pairs of *Ae. searsii* chromosomes, 1S^s^L and 6S^s^L exhibiting the largest signals. A relatively intense pAs1 signal is detected in a terminus of 4S^*s*^S and few very weak interstitial signals are observed on 1S^s^L, 3S^s^L, and 7S^s^L. Faint, dispersed, non-specific pAs1 signals are distributed in distal halves of *Ae. sharonensis* and *Ae. longissima* chromosomes, while pTa-s53 and pTa-535 did not hybridize to the chromosomes of these species.

### Analysis of Polyploid Species: Wheats

**Figure 6:**
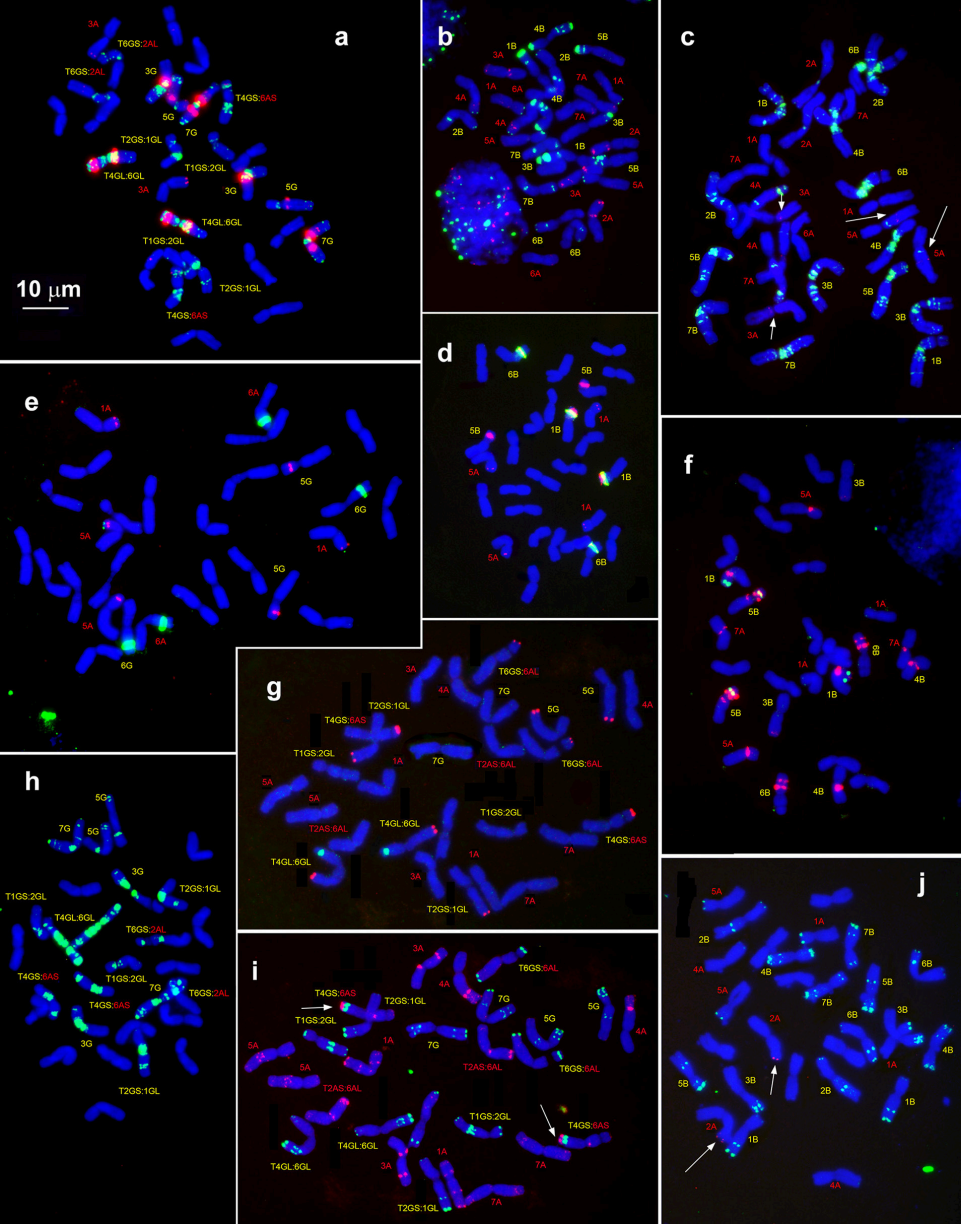
Hybridization patterns of (CTT)_10_ (**a,c,h**, green), pSc119.2 (**b,i,j**, green), pTa-535 (**c,i**, red), pTa-s53 (**b,h**, red), pAesp_SAT86 (**f,h**, red), NORs (**d,e**, green), and 5S rDNA (**d,e**, red), 5S rDNA (**f**, green); Spelt-1 (**j,g**, red) and Spelt-52 (**g**, green) on metaphase chromosomes of *T. dicoccoides*, IG 46396 **(b–d,f,j)** and *T. araraticum*, K-59940 **(a,e,h,g,i)**. Position of pTa-s53 hybridization sites on *T. dicoccoides* chromosomes **(c)**, huge cluster of pTa-535 sequence on the chromosome 4GS **(i)** and Spelt-1 site on *T. dicoccoides* chromosome 2AL **(j)** are indicated with arrows. Scale bar, 10 μm.

Differences between emmer and Timopheevii wheat are mainly due to species-specific translocations identified in both evolutionary lineages (Naranjo et al., 1987; Liu et al., 1992; Jiang and Gill, 1994a; Maestra and Naranjo, 1999; Salina et al., 2006a). The (CTT)_10_ signals on *T. araraticum* and *T. dicoccoides* chromosomes (Figures 3a,b, 6a,c,h) mainly correspond to the C-bands, whereas (GTT)_9_ forms large clusters in proximal regions of all B- and G-genome chromosomes (Figure 2, G,B; Figures 3a,b); their positions mainly overlapped with the location of (CTT)_10_ clusters. A similar pattern is also observed in *Ae. speltoides* (Figure 3f).

Although the pSc119.2 hybridization patterns in these two wheat species are distinct and species-specific, they share some similar features. As in *Ae. speltoides*, pSc119.2 signals are located in interstitial and subtelomeric regions of orthologous chromosome allowing a complete chromosome identification.

Two chromosome pairs of *T. araraticum* and *T. dicoccoides* carry major NORs (Figures 6d,e). These are 1B and 6B in *T. dicoccoides* and 6G and 6A^t^ in *T. araraticum* (transfer of NORs from 1G to 6A^t^ is due to species-specific translocation 1G-4G-6A^t^ in Timopheevii lineage, Jiang and Gill, 1993). Group 1 and 5 chromosomes of *T. dicoccoides* display eight 5S rDNA signals (Figure 6d), but only six - in *T. araraticum* (chromosomes 1A^t^, 5A^t^, and 5G, see Figure 6e). Chromosome 5S of *Ae. speltoides* shows one 5S rDNA locus, therefore 1S likely lost the 5S rDNA locus in the progenitor of *Ae. speltoides* after the formation of ancient emmer, but prior to the divergence of Timopheevii wheat.

The pAesp_SAT86 clusters are found on both A and B/G genome chromosomes (Figures 6a,f), *T. dicoccoides* and *T. araraticum* show different labeling patterns and both exhibit broad intraspecific polymorphisms (Badaeva, unpublished). A large pAesp_SAT86 signal is found on 1BS of all emmer (Figure 6f) and common wheat (Komuro et al., 2013), but it is absent on 1G of *T. timopheevii* (Figure 6a). By contrast, huge 3GL- and 7GL-located pericentromeric pAesp_SAT86 clusters are missing on the homoeologous chromosomes of emmer wheat. At the same time, similar labeling patterns were observed on 4B/4G, 5B/5G, and 6B/6G of these species.

Very weak Spelt-52 signals were seen on 1GS and large on 6GL of *T. araraticum*. The same sequence was not detectable in wild emmer. Two faint Spelt-1 signals were revealed on the chromosome pair 2A of *T. dicoccoides* (Figure 6j), whereas ten clear signals were observed on chromosomes 6A^t^S, 1GL, 4GL, 5GL, and 6GS of *T. araraticum* (Figure 6g).

Probe pTa-535 hybridized predominantly on the A-genome chromosomes of both wheat species (Figures 6b,i, red color). A large pTa-535 cluster was found on the short arm of 4G of *T. araraticum* (Figure 6i, indicated with arrows). Overlapping, small pAs1/ pTa-535 signals are detected in distal halves of 3GL and 3BL. In addition, faint pAs1 signals were found in the satellite of 1B, in the middle of 6BS and 7BL of wild emmer. *T. araraticum* carries small pAs1 loci on 5GL and 7GL and in the satellite of 6A^t^ (data not shown). Only weak pTa-s53 signals were observed on chromosomes 3AS and 5AL of *T. dicoccoides* (Figure 6c), and no hybridization was found on *T. araraticum* (Figure 6h).

### Polyploid *Aegilops*: *Ae. peregrina and Ae. kotschyi*

*Ae. peregrina* and *Ae. kotschyi* are both tetraploids with the same genome constitutions UUS^*^S^*^. Their C-banding patterns are generally similar, however, some differences in morphology and heterochromatin distribution on chromosomes 2S^*^, 4S^*^, and 7S^*^ are observed (Figure S3). According to C-banding patterns, *Ae. peregrina* carries 4S-7S^*^ translocation and therefore the S^p^-genome is originated from *Ae. longissima*. The S^k^ genome of *Ae. kotschyi* is more diverged from the S^*^-genomes of *Emarginata* species, but shares similar structure and C-banding pattern of chromosome 4S^*^ with *Ae. sharonensis* (Figures S2, S3).

**Figure 7:**
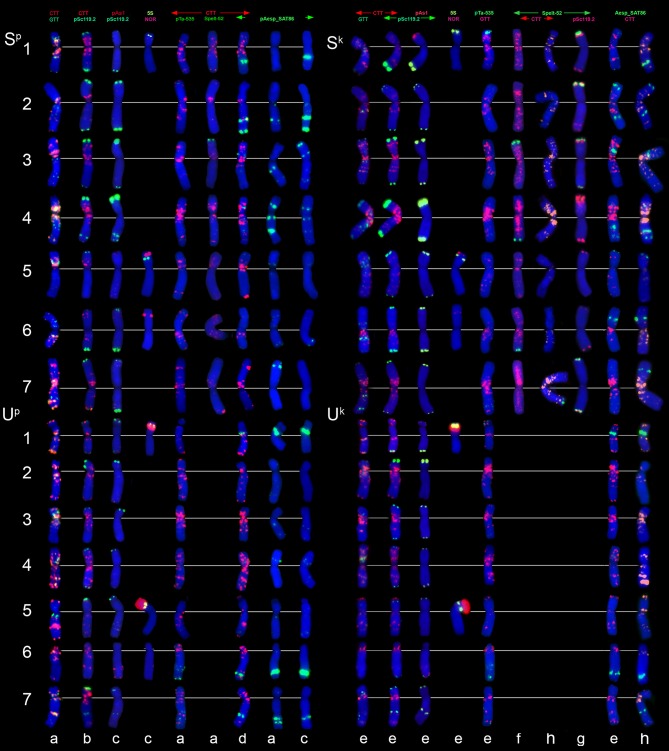
Distribution of repetitive DNA families on chromosomes of *Ae. peregrina* left side and *Ae. kotschyi* (right side of the figure): **(a)**, C11 (Nahal Mearot, Israel); **(b)**, C8 (Haifa, Carmel, Israel); **(c)**, K-61; **(d)**, C9 (Keshon, Israel); **(e)**, TA2206; **(f)**, K-91; **(g)**, hybrid *Ae. umbellulata* TU04 × *Ae. sharonensis* TH02; **(h)**, K-2905. Probe combinations are given on the top; signal color corresponds to probe name. The S-genome chromosomes are shown on the top, the U-genome–on the bottom part of the figure.

FISH with (CTT)_10_ and (GTT)_9_ probes reveals large CTT-clusters on all chromosomes, but only few weak GTT-signals on some U and S^*^-genome chromosomes of both species (Figures 3c,h). Distribution of (CTT)_10_ probe corresponds to the C-banding patterns (Figure 7, Figure S3), and dual-color FISH allows aligning of the CTT/C-banding and pSc119.2-FISH patterns (Figure 5). Positions of pSc119.2 clusters on chromosomes of the two species are similar except for 4S^*^, which carries two prominent subtelomeric signals in *Ae. kotschyi*, but one huge cluster in the short and two smaller sites in the long arm in *Ae. peregrina* (Figure 7). Labeling patterns varies between the accessions. Owing to subterminal location of pSc119.2 sites and polymorphism of labeling patterns, applicability of the pSc119.2 probe for chromosome identification of *Ae. peregrina* and *Ae. kotschyi* is limited.

Distribution of 45S and 5S rDNA loci on *Ae. peregrina* and *Ae. kotschyi* chromosomes is similar Figure 7, (NOR+5S) and corresponds to that in the parental species. Signal size of pTa71 probe (45S rDNA) on 5S^k^ and especially 6S^k^ chromosomes of *Ae. kotschyi* is significantly smaller than on the orthologous chromosomes of *Ae. peregrina*, which can be an indicative of more extensive gene loss at the respective loci.

FISH reveals similar hybridization patterns of pAesp_SAT86 probe on chromosomes of *Ae. peregrina* and *Ae. kotschyi*. According to dual-color FISH, the largest pAesp_SAT86 signals are located on chromosomes 1S^*^L (polymorphic), 2S^*^L, 1US, 6UL, and 7UL. Chromosomes 3S^*^ and 4S^*^ carry medium and 5S^*^S, 6S^*^L, and 4US – faint signals (Figure 7). Labeling patterns of chromosomes 3S^*^, 4S^*^, 6S^*^, and 7S^*^ are polymorphic. In contrast to *Ae. peregrina* and diploid *Emarginata* species, the chromosome 6S^k^ of *Ae. kotschyi* carries large pAesp_SAT86 cluster in the short arm.

The Spelt-1 repeat is not found in these tetraploid species, while Spelt-52 is revealed only in few accessions of *Ae. kotschyi* and *Ae. peregrina*. Small Spelt-52 clusters are observed on four out of seven S^*^-genome chromosome: 3S^*^S, 4S^*^L, 6S^*^L, and 7S^*^L. Number of signals varies from two to six (Figure 7), nearly half of genotypes we examined do not exhibit any hybridization. This is strictly different from what is observed in a newly synthesized hybrid *Ae. umbellulata x Ae. sharonensis*, in which 12 distinct Spelt-52 signals are observed in either one or both arms of chromosome pairs 1S^*^, 2S^*^, 3S^*^, and 7S^*^ (Figure 7g ).

Very few weak pAs1 and pTa-535 signals are located predominantly on the U-genome chromosomes of *Ae. kotschyi* and *Ae. peregrina* (Figure 7), while the pTa-s53 sequence is totally absent.

### Polyploid *Aegilops*: *Ae. vavilovii*

**Figure 8:**
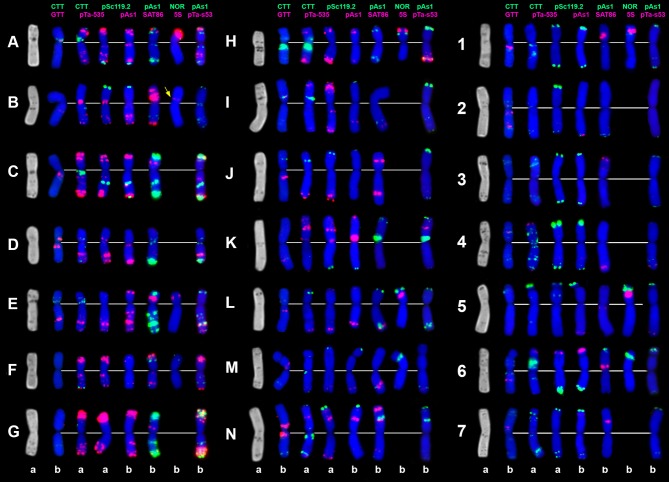
C-banding (left) and FISH patterns of *Ae. vavilovii* chromosomes: a – K-3635; b – K-3637. Chromosomes are assigned to genome D^1^ (left), X^cr^ (middle), and to the S^v^ genome (right) according to similarity with chromosomes of *Ae. crassa* (Badaeva et al., 1998) and *Ae. searsii* (Friebe et al., 1995). Probe combinations are shown on the top, signal color corresponds to probe name. Chromosomes derived from *Ae. crassa* are designated with letters (A–N); the S^v^ genome chromosomes are numbered 1–7 according to genetic nomenclature (Friebe et al., 1995).

The hexaploid species *Ae. vavilovii* with the genome constitution D^1^D^1^X^cr^X^cr^S^v^S^v^ is characterized by a medium amount of Giemsa bands. Small and medium sized bands are distributed predominantly in interstitial chromosome regions (Figure 8). Two chromosome pairs are submetacentrics with small satellites, which morphologically correspond to 5S^*^. Two other pairs are metacentrics with large satellites, which is typical for chromosome 6S^*^. The C-banding pattern of *Ae. vavilovii* is similar to the parental species: *Ae. crassa* (Badaeva et al., 1998, 2002) and *Ae. searsii* (Friebe et al., 1995; Friebe and Gill, 1996). Intraspecific variations due to chromosomal rearrangements were identified in two of the three accessions of *Ae. vavilovii*.

The distribution of (CTT)_10_ signals is generally similar to the observed C-banding patterns (Figure 8). The (GTT)_9_ probe results in distinct signals on five pairs of the S^v^ genome chromosomes and small to medium clusters on seven pairs of the D^1^ and X^cr^ genome chromosome. (GTT)_9_ signals only partially overlap with (CTT)_10_ loci (Figure 3g).

Probe pSc119.2 hybridized with all S^v^ and some X^cr^ genome chromosomes. Signals are located in subterminal chromosome regions; interstitial sites were found in the middle of 5S^v^S and in the distal region of 4S^v^L and 7S^v^L (Figure 8).

Probe pTa71 revealed eight major and eight minor NOR sites in *Ae. vavilovii*. The major NORs are located on group 5 and 6 chromosomes belonging to S^v^ and X^cr^ genomes. Minor NORs mapped on all three pairs of group 1 chromosomes and, surprisingly on 6D^1^S. Six of 5S rDNA sites are located on group 1 and 5 chromosomes. An additional, minor 5S rDNA locus is detected in the proximal region of an unknown small metacentric chromosome (Figure 8, shown with arrow).

The pAesp_SAT86 signals of different sizes were detected on many *Ae. vavilovii* chromosomes*;* the number of loci varies from one to three per chromosome (Figure 8). Distribution of pAesp_SAT86 sites on 2S^v^, 3S^v^, and 7S^v^ is different from *Ae. searsii*, while the remaining chromosomes of these genomes show similar labeling patters.

The A/D-genome-specific probes pAs1, pTa-535, and pTa-s53 hybridize mainly to the D^1^-genome and partially to X^cr^ genome chromosomes of *Ae. vavilovii* (Figure 8). The S^v^ genome possesses the lowest amount of these sequences. Small pAs1 signals were observed only in terminal regions of 4S^v^S and 6S^v^S. Neither Spelt-1, nor Spelt-52 hybridization sites were detected in *Ae. vavilovii*.

## Discussion

Karyotype analysis as a tool for studying evolutionary processes must be based on an unified chromosome nomenclature. The first classification of chromosomes according to their homoeologous relationships and genome affinities was developed for common wheat by Sears (1954), and since then it is used as standard in genetic and cytogenetic studies of the *Triticeae*. Although the nomenclature of Giemsa C-banded chromosomes is now available for many *Aegilops* species, including *Ae. speltoides, Ae. searsii, Ae. longissima*, and *Ae. peregrina* (Friebe and Gill, 1996), their correspondence to the distribution of FISH probes is not known.

In order to link C-banding and FISH patterns (Jiang and Gill, 1993) developed a method of sequential C-banding and *in situ* hybridization analysis. An alternative approach was suggested by Pedersen and Langridge (1997), who used the barley probe pHvG38 containing a GAA-satellite sequence for the identification of wheat chromosomes. Later this sequence was successfully used for the FISH analyses of wheat, barley, rye and some other cereal chromosomes (Pedersen et al., 1996; Cuadrado et al., 2000; Vrána et al., 2000; Cuadrado and Jouve, 2002; Kubaláková et al., 2005; Kato, 2011; Komuro et al., 2013; Adonina et al., 2015; Badaeva et al., 2016), however it was rarely applied for *Aegilops* species (Molnár et al., 2005, 2016; Mirzaghaderi et al., 2014).

The CTT–labeling patterns of *Aegilops* chromosomes obtained in our study basically correspond to their C-banding patterns. Therefore, we used the CTT-signals as landmark to identify chromosomes according to the genetic nomenclature. This allowed us to compare karyotypes based on chromosome homoeology and to trace chromosomal changes that could have occurred over the course of species evolution.

### *Ae. speltoides* and Polyploid Wheats Are Cytogenetically Distinct From the S^*^-Genome of Other Diploid and Polyploid *Aegilops* Species

Based on C-banding and FISH patterns it is possible to divide the S-genome chromosomes of diploid and polyploid wheat and *Aegilops* species into two distinct groups. The first one includes *Ae. speltoides* and polyploid wheat. The second contains four diploid species of the *Emarginata* sub-section and three polyploid *Aegilops*, in agreement with molecular phylogenetic analyses (Yamane and Kawahara, 2005; Golovnina et al., 2007; Goryunova et al., 2008; Salse et al., 2008; Gornicki et al., 2014; Marcussen et al., 2014; Middleton et al., 2014; Feldman and Levy, 2015). The main diagnostic features of these groups can be described as follows.

1. The satellite chromosomes of the S-genome of *Ae. speltoides* and B/G-genomes of polyploid wheats belong to homoeologous groups 1 and 6 (Dvorák et al., 1984; Friebe et al., 2000). The satellites are large and nearly equal in size (Chennaveeraiah, 1960). The satellite of *T. timopheevii* chromosome 1G is transferred to 6A^t^ as a result of a species-specific translocation (Jiang and Gill, 1994a; Rodríguez et al., 2000b; Dobrovolskaya et al., 2011). Major 45S rDNA sites are located on the short arms of group 1 and 6 chromosomes (Figure 2) (Yamamoto, 1992a,b; Jiang and Gill, 1994b; Badaeva et al., 1996b; Raskina et al., 2011; Belyayev and Raskina, 2013; Molnár et al., 2016). In addition to major NORs, Jiang and Gill (1994b) revealed minor 45S rDNA loci in the long arm of chromosome 1B of common and durum wheat, 1G of *T. timopheevii* and 1S of *Ae. speltoides*, which were never observed in other S^*^-genome *Aegilops* species. Diploid *Emarginata* species possess two pairs of satellite chromosomes assigned to genetic groups 5 and 6 (Friebe et al., 1993, 1995; Friebe and Gill, 1996); satellites significantly differ in size (Chennaveeraiah, 1960). The secondary constrictions of 5S^*^ and 6S^*^ are suppressed in polyploid *Ae. peregrina* and *Ae. kotschyi*, but are extended in hexaploid *Ae. vavilovii*. FISH with the probe pTa71 revealed major 45S rDNA sites on 5S^*^ and 6S^*^ chromosomes of diploid and polyploid *Aegilops* species, but signal sizes were significantly reduced in tetraploid *Ae. peregrina* and *Ae. kotschyi* (Figures 4, 7). Permanent minor 45S rDNA loci were present on chromosome 1S^*^, and additional minor site was detected in the terminus of 6S^*^L of all *Ae. searsii* and some *Ae. bicornis* and *Ae. longissima* accessions (Figure 5). Earlier we also found minor 45S rDNA locus in a terminus of the short arm of an unknown chromosome, probably 3S^l^, of *Ae. longissima*, accession TA1912 (Badaeva et al., 1996b). These observations are in agreement with previously published results (Yamamoto, 1992a,b; Friebe et al., 1993; Badaeva et al., 1996b, 2002, 2004).
2. The S, B, and G genomes are enriched in GTT-repeats (Figures 1–3). This microsatellite is especially abundant in proximal chromosome regions, but rarely appears in interstitial locations. The GTT-sites do not always overlap with the CTT-clusters, and proximal GTT-signals could be observed in chromosome regions lacking Giemsa C-bands. By contrast, the S^*^-genome chromosomes of *Aegilops* species show poor labeling with the (GTT)_9_ probe (Figures 3, 5). The GTT-interstitial signals mainly overlap with the (CTT)_n_ clusters (Figures 3c–e, h–j).
3. The distribution of pSc119.2 repeat in *Ae. speltoides* and the B/G genomes of wheat observed in our study (Figures 1, 2) is similar to what was reported before (Jiang and Gill, 1994a; Badaeva et al., 1996a; Schneider et al., 2003; Kubaláková et al., 2005; Salina et al., 2006b; Komuro et al., 2013) and is distinct from the S^*^-genome chromosomes of other *Aegilops* species in preferentially interstitial signal location.
4. The Spelt-1 sequence is present in the S-genome of *Ae. speltoides* (Salina et al., 1997, 2006b; Raskina et al., 2011; Belyayev and Raskina, 2013) and the B/G genomes of polyploid wheats (Salina, 2006; Salina et al., 2006b; Zoshchuk et al., 2007, 2009), but it is absent from the S^*^-genome of other diploid and polyploid *Aegilops* species.

### Different Families of Repetitive DNA Show Different Evolutionary Rates

Our data and previous findings imply that the evolutionary rate varies between different families of repetitive DNAs. Despite distinct differences between *Ae. speltoides/*polyploid wheats and other S^*^-genome *Aegilops* species in the distribution of rDNA probes, the patterns of 45S and 5S rDNA loci was highly conserved within each group. Only minor intra-and inter-specific variations were observed,

1. Regarding the appearance of minor NORs, which occur at similar positions on the orthologous chromosomes (Yamamoto, 1992a,b; Badaeva et al., 1996b), and
2. The decrease of signal size on the S^*^-genome chromosomes of tetraploid *Aegilops* species (Yamamoto, 1992a,b; Badaeva et al., 2004). Such signal reduction could be explained by uniparental elimination of genes (Shcherban et al., 2008).

The distribution of the rye-derived pSc119.2 repeat is also found to be relatively conserved within each of the two S-genome groups. This sequence with a 120 bp-long repeat unit is broadly distributed in the *Triticaea* and some *Aveneae* species and constitutes large and evolutionary old component of their genomes (Contento et al., 2005). The repeat units isolated from wheat, rye, barley and *Aegilops* species showed 70-100% similarity to each other. Nucleotide sequences of pSc119.2 repeat units are not species-specific, and one site may contain diverse members of the family (Contento et al., 2005). The authors proposed that these individual pSc119.2 sites are transferred as blocks and can be translocated within the genome resulting in position variation and site numbers. Similar was observed in our material. Most cereals, including barley (Taketa et al., 2000; Zhao et al., 2018), *Aegilops* (Badaeva et al., 1996a, 2002, 2004; Linc et al., 1999; Molnár et al., 2005, 2016), *Agropyron* (Brasileiro-Vidal et al., 2003; Li et al., 2018; Said et al., 2018), *Elytrigia* (Linc et al., 2012), *Haynaldia* (Zhang et al., 2013), possess predominantly subtelomeric pSc119.2 clusters. Therefore, a terminal location of pSc119.2 satellite family is probably a more primitive character compared to interstitial locations. Intercalary pSc119.2 sites are typical for *Ae. speltoides* (Badaeva et al., 1996a; Molnár et al., 2016), B- and G-genomes of polyploid wheats (Jiang and Gill, 1994a; Schneider et al., 2003), and rye (Cuadrado and Jouve, 2002); the rye genome being highly rearranged relative to wheat (Liu et al., 1992). Strong differences in the distribution of pSc119.2 sites in the R and S genome chromosomes suggest that transposition of this repeat proceeded in genomes of rye and *Ae. speltoides* independently, likely, after their radiation from the ancestral form.

Comparison of C-banding patterns with the distribution CTT+ GTT-microsatellite sequences shows that heterochromatin blocks detected by Giemsa staining in different *Triticum* and *Aegilops* species could have different sequence composition. Thus, *Ae. speltoides* chromosomes carry prominent proximal and telomeric C-bands and only few intercalary bands, which is considered as primitive karyotype structure (Stebbins, 1971). Only proximal bands overlap with both (CTT)_10_ and (GTT)_9_ clusters. The GTT-repeat is more abundant in these chromosomal regions. Intercalary C-bands correspond to CTT-signals, and probably they are composed by this microsatellite mainly. Neither (CTT)_10_, nor (GTT)_9_ signals were detected in telomeric heterochromatin, which is enriched in Spelt-1 and Spelt-2 repeats.

The C-banding patterns of the S^*^-genome *Emarginata* species and their polyploid derivatives are very similar to their CTT-hybridization patterns indicating that this sequence is a major component of Giemsa-positive heterochromatin. The GTT-microsatellite is present in much lower quantities, and only few C-bands contain this sequence solely. Species of this genomic group exhibit drastic differences in the content of C-positive heterochromatin. Diploid *Ae. bicornis, Ae. searsii*, and hexaploid *Ae. vavilovii* are low heterochromatic; the (CTT)_10_-signals are small and located mainly in the intercalary chromosome regions. Karyotypically *Ae. searsii* is distinct from other diploid species and its divergence was accompanied mainly by heterochromatin re-pattering visualized by Giemsa-staining and FISH with the CTT-microsatellite probe. The genomes of *Ae. sharonensis, Ae. longissima, Ae. kotschyi*, and *Ae. peregrina* are highly heterochromatic; prominent C-bands and CTT-signals are distributed in proximal and intercalary chromosome regions (Figure 5). Thus, massive amplification of the CTT-repeat occurred at the stage of radiation of *Ae. sharonensis* and *Ae. longissima*, resulting in an increase of nuclear DNA (Eilam et al., 2007) and the amount of heterochomatin.

Three tandemly repeated DNA families, pAesp_SAT86, Spelt-1, and Spelt-52 show the highest rate of evolution in the *Triticum-Aegilops* group. pAesp_SAT86 sequence is detected in all S-genome species (Figures 1, 3, 7, 8) and the B/G genomes of polyploid wheat (Figure 6). The labeling patterns are extremely variable in *Ae. speltoides* (Figure 1) and differ from polyploid wheat species which, in turn, are distinct from each other (Komuro et al., 2013; Badaeva et al., 2016). Diploid *Emarginata* species and their polyploid derivatives display species-specific patterns of pAesp_SAT86 probe (Figures 4–8). *Ae. bicornis* shows the highest degree of intraspecific pAesp_SAT86-polymorphism, while little variation has been observed in *Ae. searsii, Ae. sharonensis* (Figure 4), *Ae. kotschyi* (Figure 7), and *Ae. vavilovii* (data not shown). *Ae. bicornis* and *Ae. searsii* differ from each other and from other species of this group (Figure 5). *Ae. sharonensis* is more similar with *Ae. longissima* and *Ae. peregrina* in the distribution of pAesp_SAT86 clusters and only slightly different from *Ae. kotschyi*. The pTa-713 (homolog of pAesp_SAT86) hybridization patterns of *Ae. peregrina* reported by Zhao et al. (2016) is consistent with our results, though there are some discrepancies in chromosome designations.

The Spelt-1 repeat is found in *Ae. speltoides* and the B/G genomes of polyploid wheats. In *Ae. speltoides* it comprises nearly 2% of the nuclear genome (10^5^–10^6^ copies). The copy number of constituent sequence related to Spelt-1 is ~40–60 reduced in genomes of tetraploid wheats, and ~1200–2400 times reduced in genomes of other *Triticeae* (Pestsova et al., 1998; Salina et al., 1998; Salina, 2006). Minor amounts of Spelt-1 exist in genomes of rye, cultivated barley, most diploid and polyploid wheat as well as *Aegilops* species indicates that this sequence was already present in minor quantities in the common ancestor of the *Triticeae* (Salina et al., 1998). High homology (97–100%) of individual repetitive units implies that massive amplification of Spelt-1 repeat occurred in ancient *Ae. speltoides* after radiation from the common ancestor of the *Triticeae* (Salina et al., 1998; Salina, 2006). Spelt52 is homologous to the pAesKB52 repeat isolated earlier from *Ae. speltoides* by Anamthawat-Jonsson and Heslop-Harrison (1993). This repeat consists of monomers of two types, Spelt52.1 and Spelt52.2, which share a homologous stretch of 280 bp and have two regions without sequence similarity of 96 and 110 bp, respectively. *Ae. speltoides* displays intraspecific variation in the occurrence of Spelt52 monomer types, whereas *Ae. longissima, Ae. sharonensis*, and *Ae. bicornis* showed no interspecific variation (Salina et al., 2004a). The Spelt-52 is abundant in *Ae. speltoides* accounting for approximately 1% of nuclear genome (Anamthawat-Jonsson and Heslop-Harrison, 1993; Salina, 2006) and is also highly represented (1.0 × 10^4^ – 2.5 × 10^5^ copies) in *Ae. longissima* and *Ae. sharonensis*, but it present in minor quantities in *Ae. bicornis* and *Ae. searsii* (Salina, 2006).

Coincidently with previous findings (Salina et al., 2006b; Raskina et al., 2011; Belyayev and Raskina, 2013), we observed significant intraspecific variation of Spelt-1 and Spelt-52 labeling patterns (Figure 2). Strict differences in a ratio of Spelt-1/ Spelt-52 repeats detected between genotypes can be due to geographical origin of the material. Earlier, Raskina et al. (2011) found that the amount of Spelt-1 and, in lower extent, the Spelt-52 repeat decreases in marginal populations of *Ae. speltoides*.

Although pAs1 and pTa-535 repeats are abundant in some cereal genomes (Rayburn and Gill, 1986; Badaeva et al., 1996a; Taketa et al., 2000; Komuro et al., 2013), they are poorly represented in the S genomes of *Triticum* and *Aegilops*. Thus, we failed to detect any pAs1signals in *Ae. speltoides*, but Molnár et al. (2016) revealed small pAs1 signals on the chromosome 3S. Wheat chromosomes 3BL - 3GL and 7BL - 7GL possess pAs1 and pTa-535 clusters in similar positions (Schneider et al., 2003; Badaeva et al., 2016), although they are not detected in *Ae. speltoides*. Probably, these loci were present in the genome of ancient *Ae. speltoides*, but they were eliminated after radiation of polyploid wheats. The pAs1 and pTa-535 repeats are also poorly represented in genomes of *Emarginata* species. Two distinct interstitial pAs1 sites overlapping with either pTa-535, or with pTa-s53 loci are found in *Ae. bicornis*. pAs1 and pTa-535 are less abundant in *Ae. searsii, Ae. sharonensis* and *Ae. longissima*. Only chromosome 1S^k^ of tetraploid *Ae. kotschyi* contains a distinct pAs1/pTa-535 cluster, and these sequences are absent from the S^*^ genomes of *Ae. peregrina* and *Ae. vavilovii*.

### Evolution of the S-Genome

**Figure 9:**
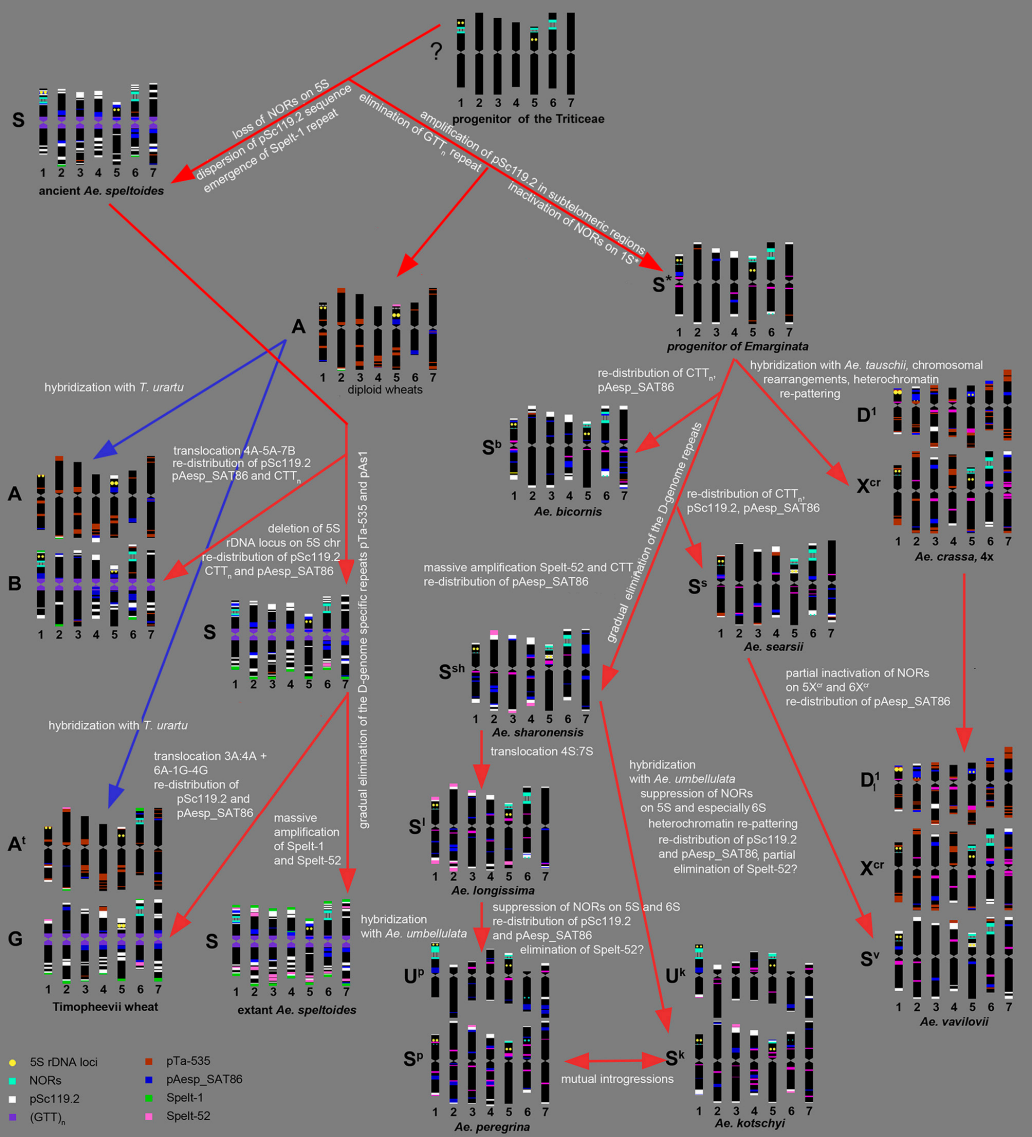
Hypothetic scheme of the S-genome evolution deduced from the results of molecular-cytogenetic analysis.

Summarizing our data and the results of other authors (Kihara, 1954; Chennaveeraiah, 1960; Kihara and Tanaka, 1970; Yen and Kimber, 1990b; Zhang and Dvorák, 1992; Zhang et al., 1992; Dvorák, 1998; Feldman and Levy, 2015), the following scenario of the S-genome evolution can be suggested (Figure 9).

According to molecular phylogeny, *Ae. speltoides* is the most distinct diploid *Aegilops*, which diverged from the common ancestor very early, prior to the split of diploid wheat and *Aegilops* species (Salse et al., 2008; Gornicki et al., 2014; Marcussen et al., 2014; Feldman and Levy, 2015). Divergence of *Ae. speltoides* from an ancestral form was not associated with major translocations, because neither meiotic analysis (Rodríguez et al., 2000a), nor microsatellite mapping (Dobrovolskaya et al., 2011) detected structural chromosmal rearrangements in the S-genome. However some genomic changes not causing linkage group perturbations did probably occur at the early stages of *Ae. speltoides* speciation. As was shown earlier, major NORs in *Triticum* and *Aegilops* species are located on group 1, 5, and 6 chromosomes (Appels et al., 1980; Appels and Honeucutt, 1986), while 5S rDNA loci are located separately from NORs in the short arms of group 1 and 5 chromosomes (Appels et al., 1980; Dvorák et al., 1989). The chromosome 5S of *Ae. speltoides* and B/G genome of polyploid wheats does not contain 45S rDNA loci, therefore the loss of respective NOR probably occurred prior to formation of ancient emmer. Other early genomic changes of ancient *Ae. speltoides* included the transposition of the pSc119.2 repeat from subtelomeric to interstitial chromosome regions and also the amplification of Spelt-1 repeat.

As mentioned above, *Ae. speltoides* and the B/G genomes of polyploid wheats are characterized by the abundance of GTT-microsatellite (Cuadrado et al., 2000; Badaeva et al., 2016), which is poorly represented in diploid wheats (Badaeva et al., 2015) and most *Aegilops* species (Figure 3). This difference can be caused by massive amplification of GTT-repeat in the ancient *Ae. speltoides*. Alternatively, this repeat could be eliminated from the progenitor of wheat and *Aegilops* species. Taking into consideration the abundance of GTT-repeat in rye and *Hordeum* (Cuadrado and Jouve, 2002, 2007; Dou et al., 2016), the second scenario seems to be more likely. The progenitor of *Ae. speltoides* probably possessed minor amounts of Spelt-52 and the D-genome specific repeats pTa-535, or pAs1, as they are still present in *Ae. speltoides* and the B/G genomes of polyploid wheats (Schneider et al., 2003; Badaeva et al., 2016; Molnár et al., 2016). However, these sequences could be of the A-genome origin, which spread to the S-genome following allopolyploidization.

The emergence of tetraploid emmer was accompanied by the species-specific translocation involving the chromosomes 4A-5A-7B (Naranjo et al., 1987; Liu et al., 1992; Maestra and Naranjo, 1999). In addition to structural chromosome rearrangements, other genetic and epigenetic changes occurred in a newly formed polyploid, including inactivation of the 45S rDNA loci on the A-genome chromosomes, re-distribution of Giemsa C-bands and repetitive DNA families on both A and B-genome chromosomes. Evolution of polyploid wheat resulted in polymorphisms of various DNA sequences and heterochromatin patterns that were described in many publications (Friebe and Gill, 1996; Schneider et al., 2003; Badaeva et al., 2016).

Subsequent evolution of *Ae. speltoides* occurred independently of polyploid emmer and was accompanied by several transposon insertions (Salse et al., 2008) and the loss of the 5S rDNA locus on the chromosome 1S, which is present in emmer and common wheat (Mukai et al., 1990), but absent in *T. timopheevii* (Badaeva et al., 2016) and modern *Ae. speltoides*. Although *T. timopheevii* derived from the same parental species as emmer, different parental genotypes were involved in the origin of these two lineages (Golovnina et al., 2007). Timopheevii wheat emerged much later, than ancient emmer - nearly 0.4 MYA (Gornicki et al., 2014) and its formation was accompanied by different species-specific translocation involving the chromosomes 1G-4G-6A^t^ + 3A^t^-4A^t^ (Jiang and Gill, 1994a; Maestra and Naranjo, 1999; Rodríguez et al., 2000b; Dobrovolskaya et al., 2011). As a result, a major NOR was translocated from chromosome 1G to 6A^t^, and a massive cluster of the A/ D-genome specific repeat pTa-535 appeared on the short arm of chromosome 4G (Figure 6g). Existence of Spelt-52 sites and a spread of Spelt-1 to most *T. timopheevii* chromosomes (Salina et al., 2006b; Zoshchuk et al., 2007; Badaeva et al., 2016) suggests a massive amplification of these sequences in *Ae. speltoides* prior to emergence of ancient *T. timopheevii*.

Results of molecular cytogenetic analysis suggest that genome re-structuring process is still ongoing in natural populations of *Ae. speltoides*. This is exemplified by intraspecific C-banding polymorphisms and diversity of labeling patterns of pAesp_SAT86, Spelt-1 and Spelt-52 probes observed in this and other studies (Belyayev and Raskina, 2011, 2013; Raskina et al., 2011), fluctuation of copy number of retrotransposons and tandem repeats, and high number of chromosomal rearrangements (Belyayev and Raskina, 2013; Shams and Raskina, 2018).

The species of the *Emarginata* group are closely related to each other (Eig, 1929; Kihara, 1954; Friebe and Gill, 1996; Kilian et al., 2011; Gornicki et al., 2014; Feldman and Levy, 2015), which is supported by their similar karyotypes (Chennaveeraiah, 1960), C-banding and pSc119.2-labeling patterns (Badaeva et al., 1996a), distribution of rDNA probes (Yamamoto, 1992a,b; Badaeva et al., 1996b). Separation of *Emarginata* species from a common ancestor was associated with inactivation of major NORs on chromosome 1S^*^ accompanied with the significant loss of 45S rDNA repeat copies. Despite similarity of pSc119.2 labeling patterns, there are obvious, but discontinuous changes in the patterns of other sequences. Our data show that most drastic changes occurred probably at the stage of radiation of *Ae. sharonensis-Ae. longissima*. These are massive amplification of Spelt-52 and CTT-repeats resulting in the gain of heterochromatin in *Ae. sharonensis* and *Ae. longissima*, leading to an approximately 12% increase of nuclear DNA content in *Ae. sharonensis/ Ae. longissima* as compared to *Ae. searsii/ Ae. bicornis* (Eilam et al., 2007). By contrast, the amount of the D-genome repeats pTa-535, pAs1 and especially pTa-s53 gradually decreased, and these sequences nearly disappeared in genomes of *Ae. sharonensis* and *Ae. longissima*. Spelt-52 patterns of *Ae. sharonensis* and *Ae. longissima* chromosomes are highly polymorphic. The similar distribution of all analyzed DNA sequences on chromosomes of *Ae. sharonensis* and *Ae. longissima* (Figures 3, 4) point to a rather recent divergence of these species, which was accompanied by the species-specific translocation 4S^*^-7S^*^ in *Ae. longissima*.

Formation of tetraploid *Ae. peregrina* and *Ae. kotschyi* did not cause significant alterations of the parental genomes. Considering the structure of chromosome 4S^*^, the S^p^-genome of *Ae. peregrina* was donated by *Ae. longissima*, while *Ae. sharonensis* or the form preceding the split of these diploids could be the source of the S^*^-genome of *Ae. kotschyi*. These data are consistent with observations of other authors (Yu and Jahier, 1992; Zhang et al., 1992; Friebe et al., 1996), however they contradict the hypothesis about the possible ancestry of *Ae. searsii* in the origin of *Ae. peregrina* (Siregar et al., 1988). Merging of U and S^*^ genomes in the tetraploid *Ae. peregrina* and *Ae. kotschyi* led to inactivation of 45S rDNA loci on the S^*^-genome chromosomes (Figure 7). Similar was also recorded in the artificial allopolyploid *Ae. umbellulata* × *Ae. sharonensis* (Shcherban et al., 2008). Significantly smaller 45S rDNA sites on *Ae. kotschyi* chromosome 6S^k^ compared to the 6S^p^ of *Ae. peregrina* evidences in favor of a higher extent of gene loss at the respective locus, which can be due to earlier origin of *Ae. kotschyi*. The assumption that *Ae. kotschyi* is an older species is also supported by higher divergence of C-banding patterns relative to the parental species.

Interestingly, *Ae. peregrina* and *Ae. kotschyi* both possess only minor quantities of the Spelt-52 repeat, which is abundant in their diploid parents. According to the analyses of artificial wheat-*Aegilops* or *Aegilops-Aegilops* hybrids, the Spelt-52 was either amplified or retained at the same level upon polyploidization (Salina et al., 2004b). Considering these results we can expect massive amplification of the Spelt-52 sequence in *Ae. peregrina* and *Ae. kotschyi* genomes. However, this is not the case. Low amount of Spelt-52 in these species can be caused by the so-called “originator effect,” if they obtained their S^*^genomes from genotype depleted with this repeat, or it can be caused by sequence elimination after formation of tetraploids.

The S^v^-genome chromosomes of *Ae. vavilovii* are very similar to the S^s^-genome chromosomes of *Ae. searsii*, which further supports their close relationships (Zhang and Dvorák, 1992; Dubkovsky and Dvorák, 1995). Our results strongly suggest that the X^cr^ genome of *Ae. vavilovii* is also the derivative of the S^*^ genome of an unknown *Emarginata* species, but not of *Ae. speltoides* as proposed by Dubkovsky and Dvorák (1995); Edet et al. (2018). Significant differences between the X^cr^ and S^s^ genomes, as well between X^cr^ and S^*^-genomes of all diploid *Emarginata* species in the C-banding and labeling patterns demonstrate that the X^cr^ genome was significantly modified during speciation.

## Conclusions

Analysis of the S-genomes of diploid and polyploid *Triticum* and *Aegilops* species using FISH with nine DNA probes, including 5S and 45S rDNA, two microsatellites and five tandem repeats showed an isolated position of *Ae. speltoides* among other *Aegilops* species. In addition, close relationships with the B and G genomes of polyploid wheats were observed, thus confirming previous molecular-phylogenetic data (Yamane and Kawahara, 2005; Petersen et al., 2006; Golovnina et al., 2007; Salse et al., 2008; Gornicki et al., 2014; Marcussen et al., 2014; Middleton et al., 2014; Bernhardt et al., 2017). The evolution of polyploid wheats was associated with different species-specific chromosome translocations and the amplification/ elimination of repeats, re-pattering or, possibly with an exchange of repetitive DNA families with the A-genome chromosomes. Evolutionary changes in the *Ae. speltoides* genome occurred independently from polyploid wheats.

Diploid *Aegilops* species of *Emarginata* group are similar, but are substantially different from *Ae. speltoides* based on C-banding and FISH patterns. The genome evolution in this group was mainly associated with an increase of high copy DNA fraction due to amplification of CTT-repeat, re-distribution of C-bands, (CTT)_n_-, (GTT)_n_-, and pAesp_SAT86-clusters, massive amplification of Spelt-52 and gradual elimination of the D-genome-specific sequences pAs1, pTa-535 and pTa-s53. These changes were more profound at the stage of divergence of *Ae. sharonensis/Ae. longissima*. Tetraploid *Ae. peregrina* and *Ae. kotschyi* originated independently from hybridization of *Ae. umbellulata* with *Ae. longissima* (*Ae. peregrina*) or *Ae. sharonensis* or its immediate precursor (*Ae. kotschyi*). The S^*^-genomes of both tetraploids show little differences to the parental species. The S^k^-genome is characterized by more modifications than the S^p^-genome, suggesting that *Ae. kotschyi* is older than *Ae. peregrina*. Chromosome introgressions recorded in some accessions of both species (Badaeva et al., 2004) can be explained by gene flow between *Ae. peregrina* and *Ae. kotschyi*.

Our study confirmed that *Ae. vavilovii* is a natural hybrid between tetraploid *Ae. crassa* and *Ae. searsii*. The similarity of C-banding and FISH patterns of *Ae. vavilovii* and corresponding parental species points to rather recent origin of this hexaploid. The assumption that the X^cr^ genome is an additional derivative of the S^*^genome obtained from an unknown or extinct species of the *Emarginata* group, which was substantially modified over the course of evolution is supported.

## Author Contributions

EB planned the research, performed and coordinate the analysis. EB and AR performed the research and wrote the paper.

### Conflict of Interest Statement

The authors declare that the research was conducted in the absence of any commercial or financial relationships that could be construed as a potential conflict of interest.

## Supplementary Material

Figure S1 Diversity of the C-banding patterns in *Aegilops speltoides* accessions: **(a)**, No1, from Turkey (provided by Dr. N. Aminov); **(b–f)**, genotypes collected from Israeli populations; **(b,c)**, C1, Technion park, Haifa; **(d)**, C2, Nahal Mearot; **(e)**, G2.46, Ramat haNadiv; **(f)**, C14, Keshon; **(g)**, no 2734 (unknown provided by Dr. B. Kilian); **(h)**, No 2 from Iran (provided by Dr. N. Aminov); **(i)**, i-570060; **(j)**, TS89, Katzir, Israel; **(k)**, PI 487233, and **(l)**, PI 487231 (from Syria); **(m)**, PI 542269 (Turkey). Arrows show unbalanced chromosome modifications.

Figure S2 Interspecific and intraspecific variation of the C-banding patterns in *Ae. bicornis* **(a1–a3)**, *Ae. searsii* **(b1–b4)**, *Ae. sharonensis* **(c1–c8)**, *and Ae. longissima* **(d1–d10)**. Accession codes: **(a1)**, TA1942; **(a2)**, TB04-3; **(a3)**, TB10-2; **(b1)**, G.7.15; **(b2)**, TE01-1; **(b3)**, G7.12; **(b4)**, IG 47619; **(c1,c2)**, C6, Keshon; **(c3)**, C5, Caesaria; **(c4)**, TH04; **(c5)**, TH01; **(c6)**, TH02; **(c7)**, C4, Atlit; **(c8)**, C7, HaBonim; **(d1)**, TL06; **(d2)**, TL01; **(d3)**, G6.77 (Sa'ad); **(d4)**, G6.58 (Tel Akko); **(d5)**, TL03; **(d6)**, C3 (HaBonim); **(d7)**, G6.32 (Nizzanim); **(d8)**, G6.55 (Zomet Shoked); **(d9)**, G17-3; **(d10)**, TL05.

Figure S3 C-banding polymorphism of *Ae. peregrina* **(a–f)** and *Ae. kotschyi* **(g–j)** chromosomes: **(a)**, TA1888; **(b)**, C11 (Nahal Mearot, Israel); **(c)**, K-61; **(d)**, C12 (Caesaria, Israel); **(e)**, C13 (Natufia, Israel); **(f)**, i-570632; **(g)**, PI 487279; **(h)**, K-91; **(i)**, TA2206; **(j)**, K-201; **(k)** K-2905. 1–7, homoeologous groups.

Table S1 List of material and their origins.

Table S2 Distribution of Spelt-52 probe on chromosomes of *Ae. longissima* and *Ae. sharonensis*.
