# Supplementary figures and images for: Evolution of the S-Genomes in Triticum-Aegilops Alliance: Evidences From Chromosome Analysis
Source: Front Plant Sci. 2018 Dec 4;9:1756. doi: 10.3389/fpls.2018.01756 (PMC6288319; doi:10.3389/fpls.2018.01756)

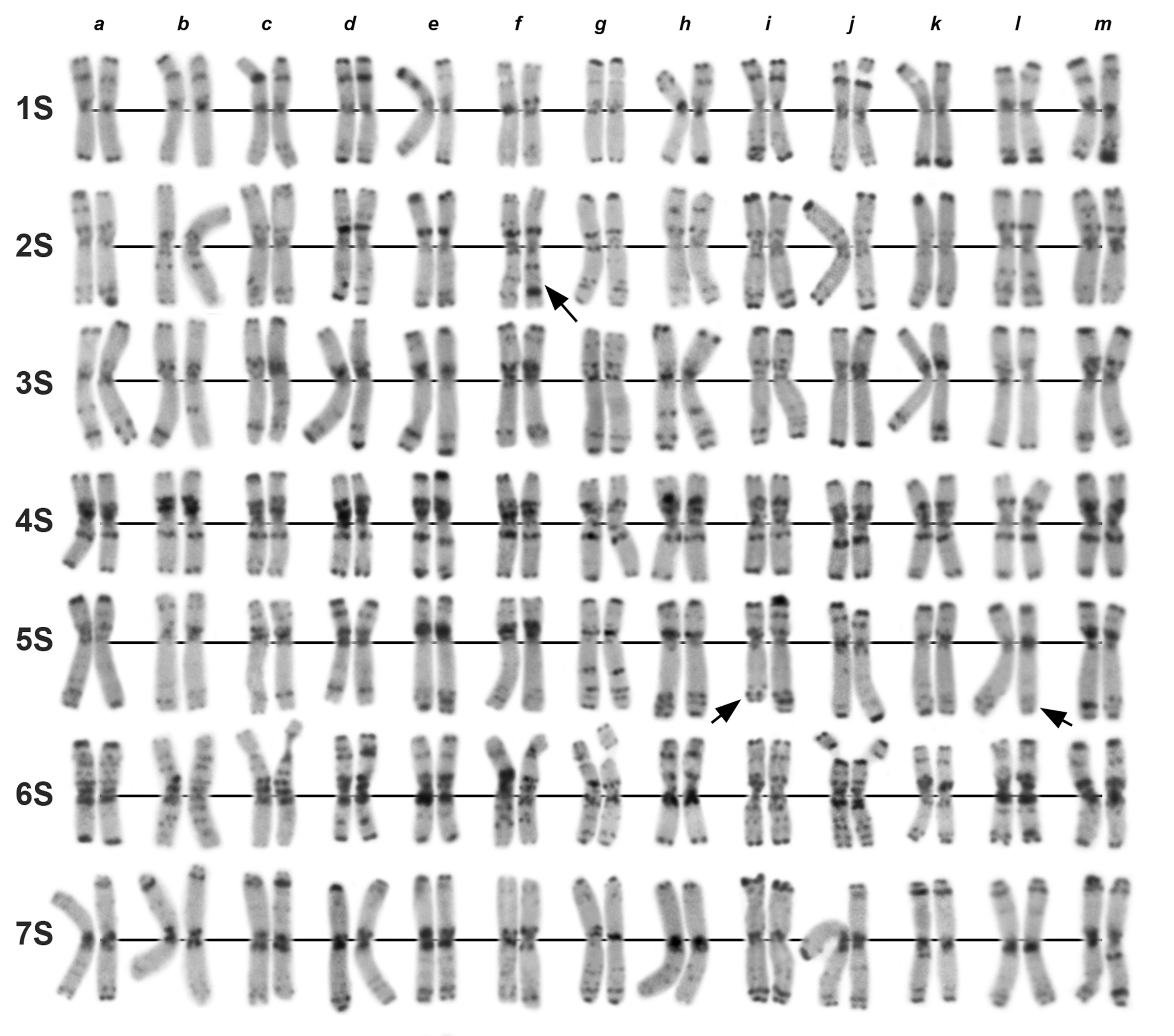

Supplement: Figure S1 — Diversity of the C-banding patterns in Aegilops speltoides accessions: (a), No1, from Turkey (provided by Dr. N. Aminov); (b–f), genotypes collected from Israeli populations; (b,c), C1, Technion park, Haifa; (d), C2, Nahal Mearot; (e), G2.46, Ramat haNadiv; (f), C14, Keshon; (g), no 2734 (unknown provided by Dr. B. Kilian); (h), No 2 from Iran (provided by Dr. N. Aminov); (i), i-570060; (j), TS89, Katzir, Israel; (k), PI 487233, and (l), PI 487231 (from Syria); (m), PI 542269 (Turkey). Arrows show unbalanced chromosome modifications. [file Image_1.TIF]

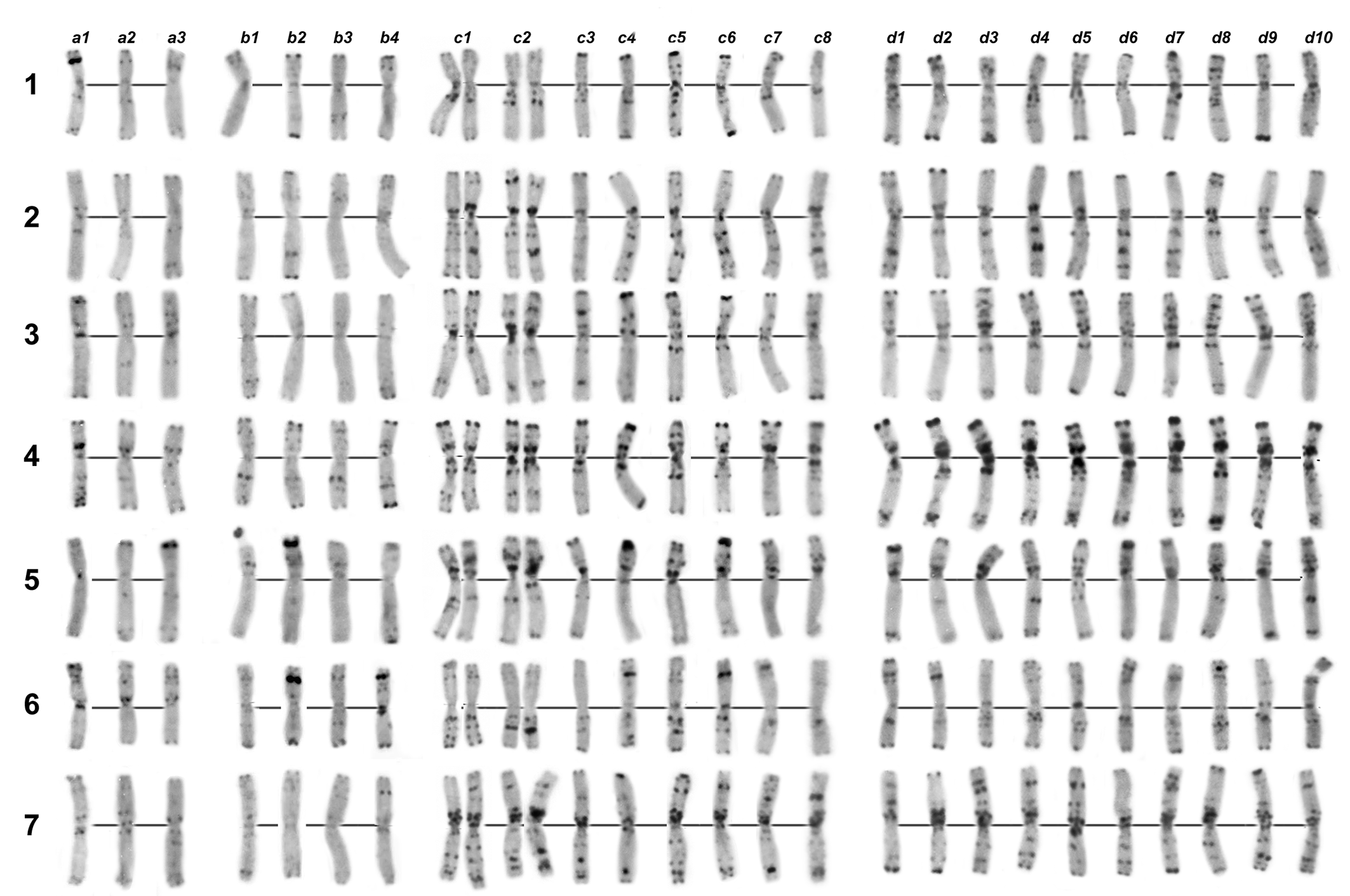

Supplement: Figure S2 — Interspecific and intraspecific variation of the C-banding patterns in Ae. bicornis (a1–a3), Ae. searsii (b1–b4), Ae. sharonensis (c1–c8), and Ae. longissima (d1–d10). Accession codes: (a1), TA1942; (a2), TB04-3; (a3), TB10-2; (b1), G.7.15; (b2), TE01-1; (b3), G7.12; (b4), IG 47619; (c1,c2), C6, Keshon; (c3), C5, Caesaria; (c4), TH04; (c5), TH01; (c6), TH02; (c7), C4, Atlit; (c8), C7, HaBonim; (d1), TL06; (d2), TL01; (d3), G6.77 (Sa'ad); (d4), G6.58 (Tel Akko); (d5), TL03; (d6), C3 (HaBonim); (d7), G6.32 (Nizzanim); (d8), G6.55 (Zomet Shoked); (d9), G17-3; (d10), TL05. [file Image_2.TIF]

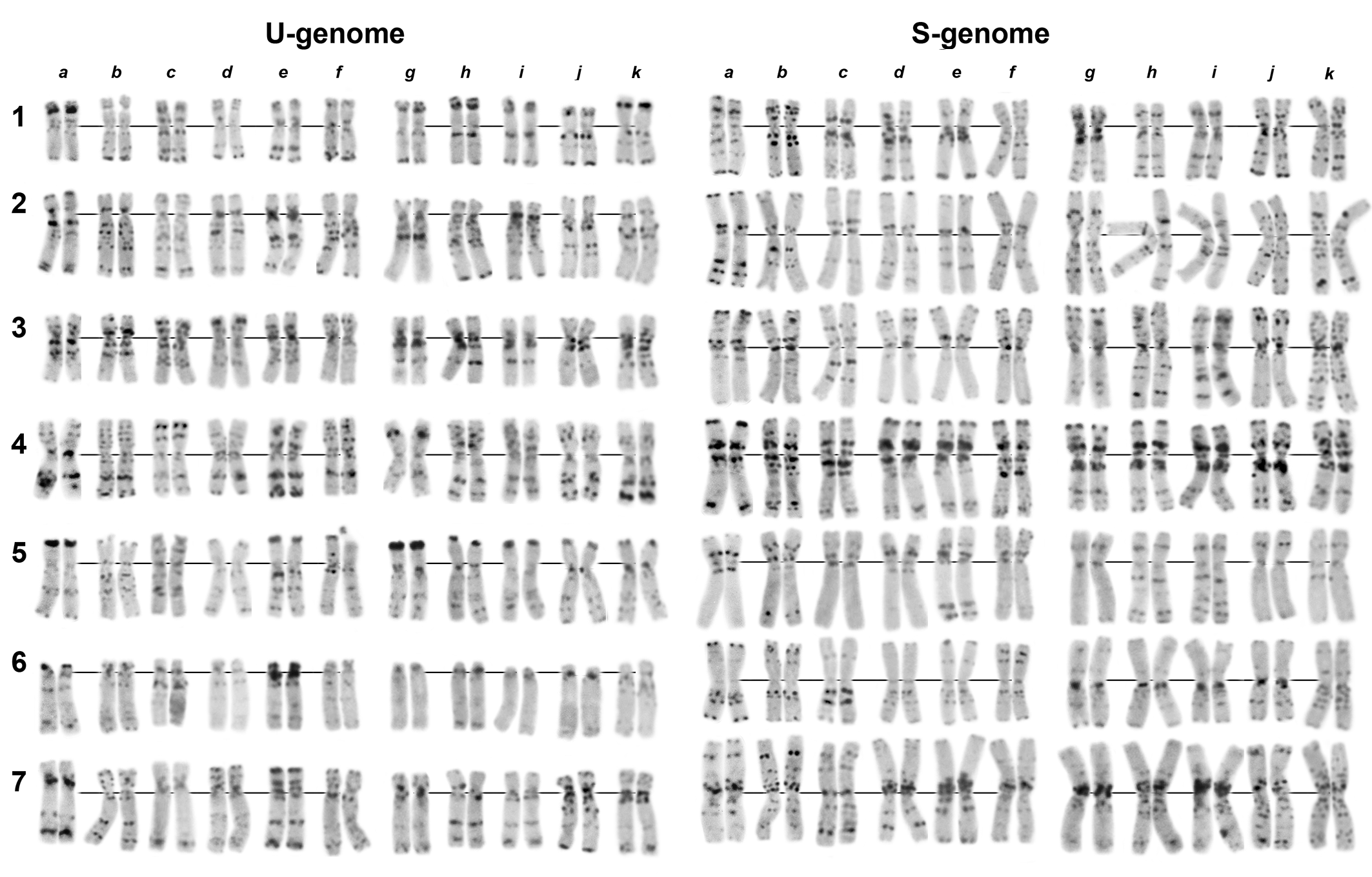

Supplement: Figure S3 — C-banding polymorphism of Ae. peregrina (a–f) and Ae. kotschyi (g–j) chromosomes: (a), TA1888; (b), C11 (Nahal Mearot, Israel); (c), K-61; (d), C12 (Caesaria, Israel); (e), C13 (Natufia, Israel); (f), i-570632; (g), PI 487279; (h), K-91; (i), TA2206; (j), K-201; (k) K-2905. 1–7, homoeologous groups. [file Image_3.TIF]
