# Supplementary material for: Evolution of the S-Genomes in Triticum-Aegilops Alliance: Evidences From Chromosome Analysis
Source: Front Plant Sci. 2018 Dec 4;9:1756. doi: 10.3389/fpls.2018.01756 (PMC6288319; doi:10.3389/fpls.2018.01756)
Supplement: Table S1 — List of material and their origins. [file Table_1.docx]

**Supplementary Tables**

Table S1 – List of material and their origins. Gene bank codes are given in Materials and Methods

| Species | Accession # | Donor* | 2n | Genome composition | County  of origin | Collection site | C-band** | FISH** |
| --- | --- | --- | --- | --- | --- | --- | --- | --- |
| *Ae. speltoides* | C1 | 6 | 14 | SS | Israel | Haifa, Technion park | 1 | 1 |
|  | C2 | 6 |  |  | Israel | Nahal Mearot | 1 | 1 |
|  | C14 | 6 |  |  | Israel | Keshon | 1 | 1 |
|  | TS89 | 3 |  |  | Israel | Katzir | 1 | 1 |
|  | G2.46 | 3 |  |  | Israel | Ramat haNadiv | 1 | 1 |
|  | K-1595 | 2 |  |  | Iran | unknown | 1 | 1 |
|  | K-3257 | 2 |  |  | Israel | unknown | - | 1 |
|  | AE 1063 | 1 |  |  | Syria | Lattakia, Shelfateh; on the road fromLattakia to Heffe | - | 1 |
|  | No 1 | - |  |  | Iran | Unknown, provided by N. Aminov | - | 1 |
|  | No 2 | - |  |  | Turkey | Unknown, provided by N. Aminov | - | 1 |
|  | PI 487231 | 4 |  |  | Syria | 1km before Roman road; between Atareb and Bab El Hawa, Aleppo Province | 1 | 1 |
|  | PI 487233 | 4 |  |  | Syria | 10km from Azaz on left road after canal in wadi valley, Aleppo Province | 1 | 1 |
|  | PI 542274 | 4 |  |  | Turkey | 20 km west of Damlacik village, 9 km north of Kahta junction | 1 | 1 |
|  | PI 542269 | 4 |  |  | Turkey | 2 km north of Gaziantep toward Yaruzeli | 1 | 1 |
|  | TA1777 | 5 |  |  | Turkey | 36 km N of Malatya | - | 2,3 |
|  | TA1783 | 5 |  |  | Israel | Technion (Israel Institute of Technology) campus; Haifa | - | 2,3 |
|  | TA1789 | 5 |  |  | Iraq | 4 km NE of Shaqlawah | - | 2,3 |
|  | TA1932 | 5 |  |  | Turkey | 4 km NW of Bismil | - | 2,3 |
| *Ae. longissima* | AE 904 | 1 | 14 | S^l^S^l^ | Israel | 3 km south from Even Yekuda | - | 1 |
|  | K-738 | 2 |  |  | unknown | unknown | - | 1 |
|  | K-907 | 2 |  |  | unknown | unknown | - | 1 |
|  | K-908 | 2 |  |  | unknown | unknown | - | 1 |
|  | K-2201 | 2 |  |  | Jordan | unknown | 1 | 1 |
|  | K-2290 | 2 |  |  | Israel | unknown | - | 1 |
|  | K-2679 | 2 |  |  | Egypt | unknown | - | 1 |
|  | TA1912 | 5 |  |  | Israel | 3 km SE of Rehovot | - | 2,3 |
|  | TA1921 | 5 |  |  | Jordan | Basin of the Jordan River | - | 2,3 |
|  | TL01-2 | 7 |  |  | Israel | Revivim, Central Negev | 1 | 7 |
|  | TL03-2 | 7 |  |  | Israel | Rehovot, coastal plain | 1 | 7 |
|  | TL05 | 7 |  |  | Israel | Nahariyyah-Rosh Haninkra Road, , Western Galilee | 1 | 7 |
|  | TL06-2 | 7 |  |  | Israel | Wadi Liman, near Rosh Hanikra, Western Galilee | 1 | 7 |
|  | G6.77 | 6 |  |  | Israel | Sa’ad | 1 | 1 |
|  | C3 | 6 |  |  | Israel | Ha Bonim | 1 | 1 |
|  | G6.32 | 6 |  |  | Israel | Nizzanim | 1 | - |
|  | G17 | 6 |  |  | Israel | No data | 1 | - |
|  | G6.55 | 6 |  |  | Israel | Zomet Shoqet | 1 | - |
|  | G6.58 | 6 |  |  | Israel | Tel Akko | 1 | - |
| *Ae. sharonensis* | K-905 | 2 | 14 | S^sh^S^sh^ | unknown | unknown | 1 | 1 |
|  | K-1584 | 2 |  |  | Israel | unknown | - | 1 |
|  | K-1675 | 2 |  |  | Israel | unknown | - | 1 |
|  | TA1995 | 5 |  |  | Turkey | From the Agean Agricultural Research Institute gene bank; Menemen; Izmir | - | 2,3 |
|  | TA1998 | 5 |  |  | Israel | 10 km N of Haifa (Hefa) to Acre (Akko) | - | 2,3 |
|  | TA 2065 | 5 |  |  |  |  | 1 | - |
|  | TH01 | 6,7 |  |  | Israel | Caesaria, coastal plain | - | 1,7 |
|  | TH02 | 7 |  |  | Israel | Naaman salt-marsh, near Acre | 1 | 7 |
|  | TH04 | 7 |  |  | unknown | No data | 1 | - |
|  | i-578030 | 2 |  |  | Israel | unknown | - | 1 |
|  | C4 | 6 |  |  | Israel | Atlit, coastal plain | 1 | 1 |
|  | C5 | 6 |  |  | Israel | Caesaria, coastal plain | 1 | 1 |
|  | C6 | 6 |  |  | Israel | Keshon | 1 | 1 |
|  | C7 | 6 |  |  | Israel | Ha Bonim | 1 | 1 |
| *Ae. searsii* | AE 1071 | 1 | 14 | S^s^S^s^ | Jordan | Jarash, 3 km S.E. Tumeiras; on the road to Bal'ama | - | 1 |
|  | AE 1072 | 1 |  |  | Jordan | Mafraq, El Buweida; on the road Bal'ama | - | 1 |
|  | AE 1076 | 1 |  |  | Jordan | Jarash, Quafqafa; 10 km N. Jarash | - | 1 |
|  | AE 1083 | 1 |  |  | Syria | Damascus, 3 km vor Tukkaya ander Straße Zabadani-Damascus | - | 1 |
|  | AE 679 | 1 |  |  | Israel | Yattir 1976, near the cultivated Pine forest | - | 1 |
|  | AE 642 | 1 |  |  | Syria | Suburbs of Gabagib | - | 1 |
|  | AE 641 | 1 |  |  | Syria | Suburbs of Rantha (Amman-Damaskus) | - | 1 |
|  | TA1837 | 5 |  |  | Palestine | E of Kurza on the Hebron (Al Khalil) Beersheba (Be'er Sheva') road; Judea | - | 2,3 |
|  | TA1841 | 5 |  |  | Palestine | Turkemiya (Tarqumiyah) Dhahiriya (Az Zahiriyah) Road | - | 2,3 |
|  | IG 47619 | 8 |  |  | Cyprus | between Meridien Limassol and Moni Misclassified (not *Ae. biuncialis*) | 1 | - |
|  | G7.12 | 6 |  |  | Israel | Mahane Ya Hir | 1 | 1 |
|  | G7.15 | 6 |  |  | Israel | Gesher Haziv | 1 | 1 |
|  | TE01-1 | 7 |  |  | Israel | unknown | 1 | - |
|  | TE17-3 | 7 |  |  | Israel | unknown | 1 | - |
| *Ae. bicornis* | AE 1079 | 1 | 14 | S^b^S^b^ | Jordan | Ma’an, 2 km W von 172-174 | - | 1 |
|  | AE 577 | 1 |  |  | unknown | unknown | - | 1 |
|  | AE 788 | 1 |  |  | Libya | Cyrenaika, Ajdabiyah, 35 kmW Marsa Brega | - | 1 |
|  | AE 106 | 1 |  |  | unknown | unknown | - | 1 |
|  | K-666 | 2 |  |  | unknown | unknown | 1 | 1 |
|  | K-1311 | 2 |  |  | unknown | unknown | - | 1 |
|  | K-1328 | 2 |  |  | Egypt | unknown | - | 1 |
|  | K-2337 | 2 |  |  | Egypt | unknown | - | 1 |
|  | TA1942 | 5 |  |  | Egypt | Marsa Matrah | 1 | 2,3 |
|  | TA1956 | 5 |  |  | unknown | unknown | 1 | 2,3 |
|  | TB04-3 | 7 |  |  | unknown | No data | 1 | - |
|  | TB10-1 | 7 |  |  | unknown | No data | 1 | - |
| *Ae. peregrina* | K-61 | 2 | 28 | U^p^U^p^S^p^S^p^ | unknown | unknown | 1,5 | 1 |
|  | K-4025 | 2 |  |  | Jordan | unknown | 1 | 1 |
|  | TA1885 | 4 |  |  | Israel | Central Coastal Plain, 10 km E of Rehovot | 5 | 1,5 |
|  | TA1891 | 5 |  |  | Israel | 3 km SE of Rehovot | 5 | 5 |
|  | TA1893 | 5 |  |  | Turkey | Opposite Eregli | 5 | 1 |
|  | TA1896 | 5 |  |  | unknown | unknown | 5 | 5 |
|  | C8 | 6 |  |  | Israel | Haifa, Carmel | 1 | 1 |
|  | C9 | 6 |  |  | Israel | Keshon | 1 | 1 |
|  | C10 | 6 |  |  | Israel | Kibbuz Ofer | 1 | 1 |
|  | C11 | 6 |  |  | Israel | Nahal Mearot | 1 | 1 |
|  | C13 |  |  |  | Israel | Natufia | 1 |  |
| *Ae. kotschyi* | TA2206 | 5 | 28 | U^k^U^k^S^k^S^k^ | Azerbaijan | Mardakan | 1,5 | 1 |
|  | TA1985 | 5 |  |  | Israel | In the garden of the Shepherd Hotel; Jerusalem | 5 | 1 |
|  | TA1979 | 5 |  |  | Israel | 25 km S of Be'er Sheva' | 5 | 1 |
|  | K-201 | 2 |  |  | unknown | unknown | 1,5 | 1,5 |
|  | K-91 | 2 |  |  | Azerbaijan | unknown | 1,5 | 1 |
|  | PI 487280 | 4 |  |  | Jordan | 6km after junction to Petra coming from Ma'an, Ma'an Province | 1 | 1 |
|  | K-2905 | 2 |  |  | Syria | unknown | 1 | 1 |
| *Ae. vavilovii* | K-3635 | 2 | 42 | D^1^D^1^X^cr^X^cr^S^v^S^v^ | Jordan | unknown | 1 | 1 |
|  | K-3637 | 2 |  |  | Jordan | unknown | 1 | 1 |
|  | TA2655 | 5 |  |  | Jordan | 132 km S of Amman, desert road at junction to Aqaba | 4 | 4 |
| *T. timopheevii* | K-46007 | 2 | 28 | GGA^t^A^t^ | Georgia | unknown | 1 | 1 |
| *T. araraticum* | K-59940 | 2 | 28 | GGA^t^A^t^ | Armenia | Ararat region, between vil. Areni and Chiva (h=1200) | 1 | 1 |
|  | K-61659 | 2 |  |  | Armenia | Ekhednadzor reg., between Agavnadzori vil. and Ekhegnadzor city | 1 | 1 |
|  | IG 116164 | 8 |  |  | Turkey | Cibeker Village or Bağbaşı (old village name) | 1 | 1 |
| *T. dicoccoides* | IG 46396 | 8 | 28 | BBAA | Syria | Few km N of Salkhad | 1 | 1 |

* gene bank code:

1 - Leibniz Institute of Plant Genetics and Crop Plant Research (IPK), Gatersleben, Germany;

2 - VIR Collection of Plant Genetic Resources, Saint Petersburg, Russia; Institute of Evolution,

3 - University of Haifa, Haifa, Israel;

4 - USDA-ARS, Aberdeen, Idaho, USA;

5 - Wheat Genetics and Genomic Resource Center, Kansas State University, Manhattan, Kansas, USA

6 - material collected in 2008 by Drs. E. Badaeva, O. Raskina and A. Belyayev in different locations of Israel;

7 - Weizmann Institute of Science, Rehovot, Israel;

8 - ICARDA, Aleppo, Syria.

** method of analysis (C-banding, FISH):

1 – analyzed in a current study;

2 – results were reported in Badaeva et al. (1996a);

3 - results were reported in Badaeva et al. (1996b);

4 - results were reported in Badaeva et al. (2002);

5 - results were reported in Badaeva et al. (2004);

6 - results were reported in Salina et al. (2006).
