# Supplementary material for: Evolution of the S-Genomes in Triticum-Aegilops Alliance: Evidences From Chromosome Analysis
Source: Front Plant Sci. 2018 Dec 4;9:1756. doi: 10.3389/fpls.2018.01756 (PMC6288319; doi:10.3389/fpls.2018.01756)
Supplement: Table S2 — Distribution of Spelt-52 probe on chromosomes of Ae. longissima and Ae. sharonensis. [file Table_2.docx]

Table S2. Distribution of Spelt-52 probe on chromosomes of different *Ae. longissima* and *Ae. sharonensis* accessions*.*

|  | 1S* |  | 2S* |  |  | 3S* |  | 4S* |  | 5S* |  | 7S* |  |
| --- | --- | --- | --- | --- | --- | --- | --- | --- | --- | --- | --- | --- | --- |
|  | S | L | S | L-1 | L-2 | S | L | S | L | S | L | S | L |
| *Ae. longissima* |  |  |  |  |  |  |  |  |  |  |  |  |  |
| K-378 | M/- | -/- |  | -/- | -/- | -/- | -/- | -/- | M/M | -/- | S/S | L/L | S/- |
| K-907/1 | M/M | -/- | -/- | -/- | -/- | -/- | -/S | -/- | -/M | -/- | S/S | -/S | -/- |
| K-907/2 | M/- | -/- | S/- | -/S | -/S | -/- | -/S | -/- | L/- | -/- | S/- | -/- | -/- |
| K-908 | M/- | -/- | M/M | -/- | -/- | -/- | L/L | -/- | M/M | -/- | S/S | L/L | -/- |
| K-2240 | -/- | -/- | -/- | -/- | -/- | -/- | -/- | -/- | -/- | -/- | S/S | M/M | -/- |
| K-2201 | -/- | -/- | -/- | -/- | -/- | -/- | -/- | -/- | -/- | -/- | S/S | S/- | -/- |
| C3 (HaBonim) | -/- | -/- | -/- | S/S | -/- | -/- | -/- | -/- | -/- | -/- | S/S | -/- | -/- |
| TL03 | -/- | -/- | -/- | S/S | -/- | -/- | M/M | S/S | -/- | -/- | -/- | -/- | -/- |
| G6.32 (Nizzarim)-1 | M/M | M/M | S/- | -/- | -/- | M/M | -/L | M/M | M/M | -/- | S/S | -/- | -/- |
| G6.32 (Nizzarim)-2 | -/- | -/- | L/L | -/- | L/- | -/- | L/M | S/- | M/- | -/- | S/S | -/- | S/- |
| AE 904 | -/- | M/M | -/- | -/S | -/- | -/- | -/- | -/- | L/L | S/- | -/- | -/- | -/- |
| AE 320 | -/- | -/- | -/M | S/S | S/S | -/- | -/- | S/S | -/- | M/M | S/S | -/- | S/S |
| AE 1077 | -/- | -/- | S/S | -/- | -/- | -/- | M/M | -/- | -/- | -/- | S/S | -/- | -/- |
| *Ae. sharonensis* |  |  |  |  |  |  |  |  |  |  |  |  |  |
| K-1584/1 | -/- | -/- | -/- | -/- | -/- | -/- | -/- | -/- | -/- | -/- | -/- | -/- | -/- |
| K-1584/2 | -/- | -/- | S/S | -/- | -/- | M/M | -/- | -/- | M/M | -/- | -/- | -/- | -/- |
| K-905 | -/- | M/M | -/- | S/S- | -/- | M/M | -/- | -/- | L/L | -/- | -/- | -/- | S/S |
| I-578030 | -/- | -/- | L/L | -/- | -/- | L/L | S/- | -/- | L/L | -/- | -/- | -/- | S/S |
| K-1675 | -/- | S/L | -/- | S/S | -/- | L/L | -/- | -/- | L/L | M/- | -/- | M/M | -/- |
| C7 (HaBonim) | -/- | M/- | -/- | S/S | -/- | -/- | M/M | -/- | -/- | -/- | S/S | -/- | S/S |
| C6 (Keshon) | -/- | S/- | -/- | S/S | S/S | -/- | -/- | -/- | -/- | -/- | -/- | -/- | -/- |
| C5 (Caesaria) | S/- | -/M | S/- | M/M | -/- | M/- | -/- | -/- | -/- | M/- | M/M | -/- | -/- |
| C4 (Atlit) | M/- | M/S | -/- | S/S | -/- | M/M | M/M | M/M | M/- | M/M | S/S | M/M | -/- |

1S* – 7S* – chromosomes; S – short arm; L – long arm. The size of hybridization sites: “S” – small; “M”- medium; “L” – large; “-“ – signal is absent. Signals on two homologs are shown for each chromosome.
